# Synthesis and Biological Evaluation of Benzo [4,5]- and Naphtho[2′,1′:4,5]imidazo[1,2-c]pyrimidinone Derivatives

**DOI:** 10.3390/biom13111669

**Published:** 2023-11-20

**Authors:** Polina Kamzeeva, Nikolai Dagaev, Sofia Lizunova, Yuri Khodarovich, Anna Sogomonyan, Anastasia Kolchanova, Vadim Pokrovsky, Vera Alferova, Alexey Chistov, Artur Eshtukov-Shcheglov, Elizaveta Eshtukova-Shcheglova, Evgeny Belyaev, Dmitry Skvortsov, Anna Varizhuk, Andrey Aralov

**Affiliations:** 1 Shemyakin-Ovchinnikov Institute of Bioorganic Chemistry, Russian Academy of Sciences, 117997 Moscow, Russia; polinabast@yandex.ru (P.K.);; 2 Department of Chemistry and Faculty of Bioengineering and Bioinformatics, Lomonosov Moscow State University, 119991 Moscow, Russia; nikolas.dagaev@yandex.ru (N.D.);; 3 Lopukhin Federal Research and Clinical Center of Physical-Chemical Medicine of Federal Medical Biological Agency, 119435 Moscow, Russia; 4 Research and Educational Resource Center for Cellular Technologies, The Peoples’ Friendship University of Russia, 117198 Moscow, Russia; 5 N.N. Blokhin Cancer Research Center, 115478 Moscow, Russia; 6 Research Institute of Molecular and Cellular Medicine, RUDN University, 117198 Moscow, Russia; 7 Lomonosov Institute of Fine Chemical Technology, MIREA-Russian Technological University, 119571 Moscow, Russia; 8 Frumkin Institute of Physical Chemistry and Electrochemistry, Russian Academy of Sciences, 119071 Moscow, Russia; 9 Moscow Institute of Physics and Technology, 141701 Dolgoprudny, Russia; 10 G4_Interact, USERN, University of Pavia, 27100 Pavia, Italy

**Keywords:** imidazopyrimidinone, anti-proliferative activity, azacarbazoles, cytostatic agent

## Abstract

Azacarbazoles have attracted significant interest due to their valuable properties, such as anti-pathogenic and antitumor activity. In this study, a series of structurally related tricyclic benzo[4,5]- and tertacyclic naphtho[2′,1′:4,5]imidazo[1,2-c]pyrimidinone derivatives with one or two positively charged tethers were synthesized and evaluated for anti-proliferative activity. Lead tetracyclic derivative **5b** with two amino-bearing arms inhibited the metabolic activity of A549 lung adenocarcinoma cells with a CC_50_ value of 3.6 μM, with remarkable selectivity (SI = 17.3) over VA13 immortalized fibroblasts. Cell-cycle assays revealed that **5b** triggers G2/M arrest without signs of apoptosis. A study of its interaction with various DNA G4s and duplexes followed by dual luciferase and intercalator displacement assays suggests that intercalation, rather than the modulation of G4-regulated oncogene expression, might contribute to the observed activity. Finally, a water-soluble salt of **5b** was shown to cause no acute toxic effects, changes in mice behavior, or any decrease in body weight after a 72 h treatment at concentrations up to 20 mg/kg. Thus, **5b** is a promising candidate for studies in vivo; however, further investigations are needed to elucidate its molecular target(s).

## 1. Introduction

**Figure 1:**
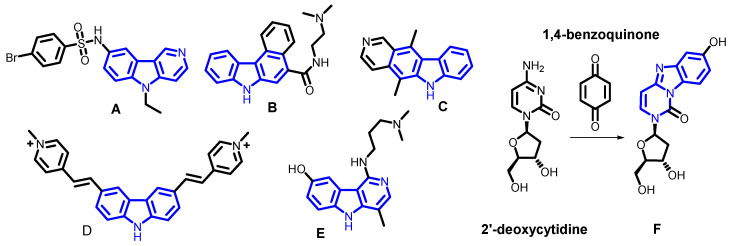
Examples of derivatives of γ-carboline (**A**,**E**) and carbazole (**B**–**D**). An example of synthesis of the adduct of 2′-deoxycytidine with 1,4-benzoquinone (**F**). Blue scaffolds are γ-carboline and carbazole.

Cancer is a life-threatening disease that causes millions of deaths worldwide annually [1]. Chemotherapy represents a significant treatment option for cancer, and it stimulates the ongoing search for new therapeutic agents [2]. Carbazole and its benzo- and aza-derivatives exhibit notable antitumor activity [3,4,5,6,7,8]. They demonstrate a variety of mechanisms of action, including the inhibition of tubulin polymerization [9,10] and DNA-binding enzymes [11,12,13] (Figure 1A–C). A carbazole derivative, BMVC, possesses an ability to stabilize telomeric G-quadruplex (G4) structures and thus inhibits telomerase activity, which is responsible for cell immortalization [11,14] (Figure 1D). Carbazole-based derivatives are also able to stabilize G4s in the cMyc promoter, resulting in the inhibition of oncogene transcription [15]. Compounds with the γ-carboline scaffold intercalate into duplex DNA and regulate the activity of topoisomerase II [12,13,16,17] (Figure 1E). The cellular targets of other antitumor carbazole-based derivatives remain unknown and are still to be investigated [18].

More than 30 years ago, an adduct of 2′-deoxycytidine with 1,4-benzoquinone was synthesized during the investigation of the cancerogenic properties of benzene [19] (Figure 1F). However, a biological evaluation of this heteroaromatic scaffold was not performed. Taking into account the intriguing antitumor activity of γ-carbolines, we decided to evaluate this property for a set of compounds with a benzo[4,5]imidazo[1,2-c]pyrimidinone scaffold. Since DNA fragments are considered the main target of most γ-carbolines, we designed compounds in a way that allows them to effectively interact with DNA structures. In particular, the chosen planar aromatic system, supposed to stabilize duplex and G4 DNA structures via stacking interactions, was expanded to naphtho[2′,1′:4,5]imidazo[1,2-c]pyrimidinone in order to enhance stabilization efficiency [20,21]. Moreover, basic side chains were introduced into the compounds to provide electrostatic interaction with the negatively charged DNA sugar-phosphate backbone [20,21]. In addition to the unmodified amino group, guanidino- and dimethylamino-containing substituents were proposed due to their higher basicity. The choice of the aforementioned modifications is supported by their successful application in the design of carbazole- and γ-carboline-based derivatives with antitumor activity [5,12,16]. Thus, we synthesized a series of benzo[4,5]- and naphtho[2′,1′:4,5]imidazo[1,2-c]pyrimidinones with one or two positively charged tethers. Their cytotoxicity and selectivity of action was evaluated on cell lines of cancerous and non-cancerous etiology, and possible DNA-related mechanisms of action were investigated.

## 2. Materials and Methods

1,8-Diazabicyclo[5.4.0]undec-7-ene (DBU) and 37% aqueous CH_2_O were purchased from Thermo Fisher Scientific (Madison, WI, USA). 1,4-Benzoquinone, 1,4-naphthoquinone, methyl 2-bromoacetate, ethane-1,2-diamine, 1H-pyrazole-1-carboxamidine hydrochloride, and sodium cyanoborohydride were obtained from Sigma-Aldrich (St. Louis, MO, USA). Solvents were purchased from commercial sources and used without further purification, except for CH_2_Cl_2_, which was distilled over calcium hydride. Thin layer chromatography (TLC) was performed on plates (Merck, Darmstadt, Germany) pre-coated with silica gel (60 mm, F_254_) and visualized using UV light (254 and 365 nm). Column chromatography (CC) was performed on silica gel (0.040–0.063 mm, Merck, Germany). Volatiles were evaporated on a Heidolph Hei-VAP Precision ML/G3B rotary evaporator (Schwabach, Germany). ^1^H and ^13^C spectra were recorded on a Bruker Avance III 600 spectrometer (Bruker, Rheinstetten, Germany) at 600 and 150 MHz, respectively, or on a Bruker Fourier 300 (Bruker, Rheinstetten, Germany) at 300 and 75 MHz, respectively, at 303 K. Chemical shifts are reported in δ (ppm) units using residual 1H signals from deuterated solvents as references. Multiplicity is reported using the following abbreviations: s (singlet), d (doublet), t (triplet), m (multiplet), and br (broad). Coupling constants (J) are given in Hz. HRMS analysis was performed using a Thermo LTQ Orbitrap XL ion trap instrument in positive ion mode (ESI+, 150–2000 au). The preparative HPLC purification of compounds **6a**,**b** and **7a**,**b** was performed on an Interchim Puriflash 4250 preparative chromatograph using a VDShpere 100 C18-E 250 × 20 mm, 5 µm column. CH_3_CN (with 0.1% TFA) and aq. TFA (0.1%) were used as eluents. UV-vis detection was performed at 210 nm, 254 nm, 350 nm, and 400 nm. A linear gradient from 5 to 95% of CH_3_CN in 10 min, followed by 4 min of 95% CH_3_CN at 20 mL/min flow rate, was used. Fractions containing the target compound were collected, organic solvent was removed in vacuo, and water was lyophilized. The HPLC purity analysis was performed using an Agilent 1260 Infinity II instrument. LC parameters: flow 1 mL/min on a Macherey Nagel Nucleodur Gravity column C18 EC (4.6 × 250 mm, 5 μm) and gradient elution method. Mobile phases: A—aq. TFA (0.1%), B—CH_3_CN (with 0.1% TFA). Gradient program: 0–10 min from 5 to 95% B, 10–14 min 95% B. UV detection wavelengths: 225, 254, 350, and 400 nm. Methyl 4-amino-2-oxo-1(2H)-pyrimidine acetate **1** was prepared according to the reported procedure [22].

### 2.1. Chemical Synthesis

#### 2.1.1. Methyl 2-(7-Hydroxy-1-oxobenzo[4,5]imidazo[1,2-c]pyrimidin-2(1H)-yl)acetate **2a**

To a suspension of methyl 4-amino-2-oxo-1(2H)-pyrimidine, acetate **1** (390 mg, 2.12 mmol) in 0.1 M sodium acetate buffer (50 mL, pH 4.5), 1,4-benzoquinone (960 mg, 8.90 mmol, 4.2 eq.) was added, and the resulting mixture was stirred at 37 °C for 24 h. The mixture was evaporated in vacuo, co-evaporated with CH_3_CN (2 × 10 mL), and subjected to column chromatography with dry loading on silica gel (0→3% CH_3_OH in CH_2_Cl_2_), yielding **2a** as a brownish amorphous solid (347 mg, 1.27 mmol, 60%).

^1^H NMR (300 MHz, DMSO-*d_6_*): δ 9.63 (br s, 1H), 7.72 (d, J = 2.4 Hz, 1H), 7.61 (d, J = 7.8 Hz, 1H), 7.56 (d, J = 8.7 Hz, 1H), 6.97 (dd, J = 8.7 Hz, J = 2.4 Hz, 1H), 6.69 (d, J = 7.8 Hz, 1H), 4.83 (s, 2H), 3.74 (s, 3H). ^13^C NMR (75 MHz, DMSO-*d_6_*): δ 168.4, 154.2, 147.1, 137.0, 136.2, 130.0, 118.8, 115.5, 114.9, 100.3, 97.2, 52.3, 49.2. HRMS (ESI) *m*/*z*: calcd for C_13_H_12_N_3_O_4_^+^ [M+H]^+^: 274.0822; found 274.0820.

#### 2.1.2. Methyl 2-(5-Hydroxy-11-oxonaphtho[2′,1′:4,5]imidazo[1,2-c]pyrimidin-10(11H)-yl)acetate **2b**

To a suspension of methyl 4-amino-2-oxo-1(2H)-pyrimidine, acetate **1** (765 mg, 4.18 mmol) in a mixture of 0.1 M sodium acetate buffer (100 mL, pH 4.5) and C_2_H_5_OH (50 mL), 1,4-naphthoquinone (2.64 g, 16.7 mmol, 4.0 eq.) was added, and the resulting mixture was stirred at 50 °C for 7 days. The mixture was evaporated in vacuo, co-evaporated with CH_3_CN (2 × 10 mL), and subjected to column chromatography with dry loading on silica gel (0→3% CH_3_OH in CH_2_Cl_2_), yielding **2b** as a brown amorphous solid (284 mg, 0.88 mmol, 21%).

^1^H NMR (600 MHz, DMSO-*d_6_*): δ 10.40 (s, 1H), 8.50 (d, J = 8.2 Hz, 1H), 8.29 (d, J = 8.2 Hz, 1H), 7.89 (s, 1H), 7.69 (ddd, J = 1.2 Hz, J = 6.9 Hz, J = 8.2 Hz, 1H), 7.59 (d, J = 7.8 Hz, 1H), 7.58 (ddd, J = 1.2 Hz, J = 6.9 Hz, J = 8.2 Hz, 1H), 6.84 (d, J = 7.8 Hz, 1H), 4.87 (s, 2H), 3.75 (s, 3H). ^13^C NMR (150 MHz, DMSO-*d_6_*): δ 168.5, 150.5, 147.4, 145.7, 135.4, 132.6, 127.0, 126.1, 125.6, 124.9, 123.6, 123.0, 121.9, 97.8, 95.9, 52.3, 49.4. HRMS (ESI) *m*/*z*: calcd for C_17_H_14_N_3_O_4_^+^ [M+H]^+^: 324.0979; found 324.0978.

#### 2.1.3. General Procedure for the Preparation of Ligands **3** with One Amino-Containing Tether

To a solution of **2** (0.1 mmol) in CH_3_OH (5 mL), ethane-1,2-diamine (67 μL, 1.0 mmol, 10 eq.) was added, and the reaction mixture was stirred at 50 °C for 48 h. The mixture was evaporated to dryness, triturated with CH_2_Cl_2_ (5 mL), and filtered, affording **3**.

N-(2-Aminoethyl)-2-(7-hydroxy-1-oxobenzo[4,5]imidazo[1,2-c]pyrimidin-2(1H)-yl)acetamide **3a**

Starting from **2a**, the title compound was obtained with a yield of 83% as a brownish amorphous solid. ^1^H NMR (300 MHz, DMSO-*d_6_*): δ 8.21 (t, J = 5.6 Hz, 1H), 7.72 (d, J = 2.4 Hz, 1H), 7.54 (d, J = 7.8 Hz, 1H), 7.50 (d, J = 8.7 Hz, 1H), 6.94 (dd, J = 8.7 Hz, J = 2.4 Hz, 1H), 6.60 (d, J = 7.8 Hz, 1H), 4.59 (s, 2H), 3.14 (dt, J = 6.4 Hz, J = 5.6 Hz, 2H), 2.64 (t, J = 6.4 Hz, 2H). ^13^C NMR (75 MHz, DMSO-*d_6_*): δ 166.6, 153.8, 147.5, 147.4, 137.1, 137.1, 130.3, 118.9, 114.5, 100.3, 97.0, 50.3, 41.7, 40.8. HRMS (ESI) *m*/*z*: calcd for C_14_H_16_N_5_O_3_^+^ [M+H]^+^: 302.1248; found 302.1247.

N-(2-Aminoethyl)-2-(5-hydroxy-11-oxonaphtho[2′,1′:4,5]imidazo[1,2-c]pyrimidin-10(11H)-yl)acetamide **3b**

Starting from **2b,** the title compound was obtained with a yield of 80% as a brownish amorphous solid. ^1^H NMR (600 MHz, DMSO-*d_6_*): δ 8.49 (d, J = 8.0 Hz, 1H), 8.30–8.24 (m, 2H), 7.91 (s, 1H), 7.68 (dd, J = 8.0 Hz, J = 7.0 Hz, 1H), 7.57 (dd, J = 8.3 Hz, J = 7.0 Hz, 1H), 7.53 (d, J = 7.2 Hz, 1H), 6.78 (d, J = 7.2 Hz, 1H), 4.64 (s, 2H), 3.18–3.09 (m, 2H), 2.66–2.62 (m, 2H). ^13^C NMR (150 MHz, DMSO-*d_6_*): δ 166.6, 150.1, 147.6, 146.1, 135.9, 133.0, 126.8, 126.1, 125.7, 124.6, 123.5, 123.0, 121.9, 97.5, 96.1, 50.5, 41.9, 40.9. HRMS (ESI) *m*/*z*: calcd for C_18_H_18_N_5_O_3_^+^ [M+H]^+^: 352.1404; found 352.1404.

#### 2.1.4. General Procedure for the Preparation of Intermediates **4**

To a solution of **2** (0.7 mmol) in CH_2_Cl_2_ (20 mL), DBU (210 μL, 1.40 mmol, 2.0 eq.) was added at room temperature, followed by the addition of methyl 2-bromoacetate (100 μL, 1.05 mmol, 1.5 eq.). The reaction mixture was kept at room temperature for 1 h, and then DBU (210 μL, 1.40 mmol, 2.0 eq.) and methyl 2-bromoacetate (100 μL, 1.05 mmol, 1.5 eq.) were added sequentially. The addition was repeated once. After 1 h, the organic layer was washed with 5% aqueous citric acid solution (2 × 20 mL) and evaporated in vacuo. The residue was purified using column chromatography on silica gel (0→1% CH_3_OH in CH_2_Cl_2_), yielding **4**.

Methyl 2-(7-(2-methoxy-2-oxoethoxy)-1-oxobenzo[4,5]imidazo[1,2-c]pyrimidin-2(1H)-yl)acetate **4a**

Starting from **2a**, the title compound was obtained with a yield of 43% as a brownish amorphous solid. ^1^H NMR (300 MHz, CDCl_3_): δ 7.92 (d, J = 2.5 Hz, 1H), 7.75 (d, J = 8.9 Hz, 1H), 7.22 (m, 2H), 6.77 (d, J = 7.8 Hz, 1H), 4.74 (s, 2H), 4.74 (s, 2H), 3.83 (s, 3H), 3.82 (s, 3H). ^13^C NMR (75 MHz, CDCl_3_): δ 169.0, 167.6, 155.5, 147.7, 147.1, 136.6, 136.5, 130.1, 119.2, 116.5, 100.0, 98.1, 66.0, 53.0, 52.3, 49.7. HRMS (ESI) *m*/*z*: calcd for C_16_H_16_N_3_O_6_^+^ [M+H]^+^: 346.1034; found 346.1033.

Methyl 2-(5-(2-methoxy-2-oxoethoxy)-11-oxonaphtho[2′,1′:4,5]imidazo[1,2-c]pyrimidin-10(11H)-yl)acetate **4b**

Starting from **2b**, the title compound was obtained with a yield of 48% as a brownish amorphous solid. ^1^H NMR (600 MHz, DMSO-*d_6_*): δ 8.53 (d, J = 8.2 Hz, 1H), 8.36 (d, J = 8.2 Hz, 1H), 7.79 (s, 1H), 7.75 (t, J = 7.6 Hz, 1H), 7.66 (t, J = 7.6 Hz, 1H), 7.64 (d, 7.8 Hz, 1H), 6.88 (d, J = 7.8 Hz, 1H), 5.08 (s, 2H), 4.88 (s, 2H), 3.76 (s, 3H), 3.75 (s, 3H). ^13^C NMR (150 MHz, DMSO-*d_6_*): δ 168.9, 168.5, 150.2, 147.4, 146.6, 135.8, 134.4, 127.5, 125.6, 125.5, 125.3, 123.7, 122.6, 122.0, 98.0, 94.3, 65.5, 52.4, 51.9, 49.5. HRMS (ESI) *m*/*z*: calcd for C_20_H_18_N_3_O_6_^+^ [M+H]^+^: 396.1190; found 396.1188.

#### 2.1.5. General Procedure for the Preparation of Derivatives **5** with Two Amino-Containing Tethers

To a solution of **4** (0.3 mmol) in CH_3_OH (20 mL), ethane-1,2-diamine (400 μL, 6.0 mmol) was added, and the reaction mixture was stirred at 50 °C for 48 h. The mixture was evaporated to dryness, triturated with CH_2_Cl_2_ (10 mL), and filtered, affording **5**.

N-(2-Aminoethyl)-2-(7-(2-((2-aminoethyl)amino)-2-oxoethoxy)-1-oxobenzo[4,5]imidazo[1,2-c]pyrimidin-2(1H)-yl)acetamide **5a**

Starting from **4a**, the title compound was obtained with a yield of 91% as a beige amorphous solid. HPLC rt 5.5 min. ^1^H NMR (300 MHz, DMSO-*d_6_*): δ 8.32 (t, J = 5.2 Hz, 1H), 8.20 (t, J = 5.6 Hz, 1H), 7.89 (d, J = 2.4 Hz, 1H), 7.67 (d, J = 8.8 Hz, 1H), 7.59 (d, J = 7.8 Hz, 1H), 7.18 (dd, J = 8.8 Hz, J = 2.4 Hz, 1H), 6.66 (d, J = 7.8 Hz, 1H), 4.63 (s, 2H), 4.55 (s, 2H), 3.27–3.14 (m, 4H), 2.73–2.64 (m, 4H). ^13^C NMR (75 MHz, DMSO-*d_6_*): δ 167.7, 166.7, 154.1, 148.4, 147.3, 138.6, 137.8, 130.0, 119.0, 114.5, 99.9, 97.0, 67.7, 50.4, 48.4, 40.8, 40.7, 40.2. HRMS (ESI) *m*/*z*: calcd for C_18_H_24_N_7_O_4_^+^ [M+H]^+^: 402.1884; found 402.1882.

N-(2-Aminoethyl)-2-(5-(2-((2-aminoethyl)amino)-2-oxoethoxy)-11-oxonaphtho[2′,1′:4,5]imidazo[1,2-c]pyrimidin-10(11H)-yl)acetamide **5b**

Starting from **4b**, the title compound was obtained with a yield of 90% as a beige amorphous solid. HPLC rt 6.3 min. ^1^H NMR (600 MHz, DMSO-*d_6_*): δ 8.53 (d, J = 8.1 Hz, 1H), 8.48 (d, J = 8.3 Hz, 1H), 8.27 (t, J = 5.2 Hz, 1H), 8.22 (t, J = 5.4 Hz, 1H), 7.88 (s, 1H), 7.75 (dd, J = 8.1 Hz, J = 7.0 Hz, 1H), 7.65 (dd, J = 8.3 Hz, J = 7.0 Hz, 1H), 7.59 (d, J = 7.7 Hz, 1H), 6.82 (d, J = 7.7 Hz, 1H), 4.77 (s, 2H), 4.66 (s, 2H), 3.24–3.17 (m, 2H), 3.15–3.10 (m, 2H), 2.66 (t, J = 6.3 Hz, 2H), 2.62 (t, J = 5.9 Hz, 2H). ^13^C NMR (150 MHz, DMSO-*d_6_*): δ 167.2, 166.5, 150.3, 147.5, 146.9, 136.5, 134.3, 127.3, 125.5, 125.5, 125.3, 123.6, 123.0, 121.9, 97.4, 94.7, 67.8, 50.6, 42.3, 41.7, 41.1, 41.0. HRMS (ESI) *m*/*z*: calcd for C_22_H_26_N_7_O_4_^+^ [M+H]^+^: 452.2041; found 452.2040.

#### 2.1.6. General Procedure for the Preparation of Di-Trifluoroacetate Salt of Derivatives **6** Bearing Two Dimethylamino-Containing Tethers

To a solution of **5** (0.05 mmol), HOAc (60 μL) and 37% aqueous CH_2_O (20 μL, 0.25 mmol) in MeOH (5 mL), NaCNBH_3_ (19.0 mg, 0.3 mmol) was added in small portions with vigorous stirring at 0 °C. After stirring for 2 h at room temperature, MeOH was evaporated. The residue was diluted with water (4 mL) and purified using preparative HPLC, yielding **6**.

Ditrifluoroacetate salt of N-(2-(dimethylamino)ethyl)-2-(7-(2-((2-(dimethylamino)ethyl)amino)-2-oxoethoxy)-1-oxobenzo[4,5]imidazo[1,2-c]pyrimidin-2(1H)-yl)acetamide **6a**

Starting from **5a**, the title compound was obtained with a yield of 38% as a pale yellow amorphous solid. HPLC rt 5.7 min. ^1^H NMR (600 MHz, DMSO-*d_6_*): δ 8.59 (t, J = 5.8 Hz, 1H), 8.48 (t, J = 5.8 Hz, 1H), 7.87 (d, J = 2.5 Hz, 1H), 7.69 (d, J = 8.8 Hz, 1H), 7.66 (d, J = 7.8 Hz, 1H), 7.21 (dd, J = 8.8 Hz, J = 2.5 Hz, 1H), 6.69 (d, J = 7.8 Hz, 1H), 4.70 (s, 2H), 4.60 (s, 2H), 3.51 (dt, J = 5.8 Hz, J = 6.0 Hz, 1H), 3.47 (dt, J = 5.8 Hz, J = 6.0 Hz, 1H), 3.14 (t, J = 6.0 Hz, 1H), 3.10 (t, J = 6.0 Hz, 1H), 2.75 (s, 6H), 2.74 (s, 6H). ^13^C NMR (150 MHz, DMSO-*d_6_*): δ 168.3, 167.3, 154.1, 148.4, 147.4, 138.7, 137.8, 130.0, 119.2, 114.6, 99.8, 97.2, 67.6, 55.8, 55.5, 42.4, 42.4, 34.2, 33.8. HRMS (ESI) *m*/*z*: calcd for C_22_H_32_N_7_O_4_^+^ [M+2H]^2+^: 229.6292; found 229.6291.

Ditrifluoroacetate salt of N-(2-(dimethylamino)ethyl)-2-(5-(2-((2-(dimethylamino)ethyl)amino)-2-oxoethoxy)-11-oxonaphtho[2′,1′:4,5]imidazo[1,2-c]pyrimidin-10(11H)-yl)acetamide **6b**

Starting from **5b**, the title compound was obtained with a yield of 33% as a pale yellow amorphous solid. HPLC rt 6.5 min. ^1^H NMR (600 MHz, DMSO-*d_6_*): δ 8.54 (d, J = 8.0 Hz, 1H), 8.47 (d, J = 8.4 Hz, 1H), 8.25 (t, J = 5.2 Hz, 1H), 8.15 (t, J = 5.4 Hz, 1H), 7.89 (s, 1H), 7.76 (dd, J = 8.0 Hz, J = 7.0 Hz, 1H), 7.66 (dd, J = 8.4 Hz, J = 7.0 Hz, 1H), 7.60 (d, J = 7.7 Hz, 1H), 6.83 (d, J = 7.7 Hz, 1H), 4.76 (s, 2H), 4.66 (s, 2H), 3.34–3.29 (m, 2H), 3.25–3.20 (m, 2H), 2.46 (t, J = 6.6 Hz, 2H), 2.38 (t, J = 6.5 Hz, 2H), 2.23 (s, 6H), 2.21 (s, 6H). ^13^C NMR (150 MHz, DMSO-*d_6_*): δ 167.1, 166.4, 150.1, 147.5, 146.9, 136.5, 134.4, 127.3, 125.5, 125.5, 125.3, 123.6, 122.9, 121.9, 97.4, 94.8, 67.8, 57.8, 57.6, 50.5, 44.9, 44.7, 36.7, 36.1. HRMS (ESI) *m*/*z*: calcd for C_26_H_34_N_7_O_4_^+^ [M+H]^+^: 508.2667; found 508.2664.

#### 2.1.7. General Procedure for the Preparation of Trifluoroacetate Salt of Derivatives **7** Bearing Two Guanidino-Containing Tethers

To a solution of **5** (0.05 mmol) in DMSO (1.5 mL), DIPEA (35 μL, 0.2 mmol, 4 eq.), followed by 1H-pyrazole-1-carboxamidine hydrochloride (22.0 mg, 0.15 mmol, 3 eq.), was added. The reaction mixture was heated for 3 h at 60 °C, cooled to room temperature, diluted with water (2.5 mL), and purified using preparative HPLC, yielding **7**.

Ditrifluoroacetate salt of N-(2-guanidinoethyl)-2-(7-(2-((2-guanidinoethyl)amino)-2-oxoethoxy)-1-oxobenzo[4,5]imidazo[1,2-c]pyrimidin-2(1H)-yl)acetamide **7a**

Starting from **5a**, the title compound was obtained with a yield of 47% as a yellowish amorphous solid. HPLC rt 5.8 min. ^1^H NMR (600 MHz, DMSO-*d_6_*): δ 9.31 (br s, 1H), 9.25 (br s, 1H), 8.77 (m, 1H), 8.46 (m, 1H), 7.94–7.72 (m, 4H), 7.88 (d, J = 2.4 Hz, 1H), 7.67 (d, J = 8.8 Hz, 1H), 7.61 (d, J = 7.6 Hz, 1H), 7.18 (dd, J = 8.8 Hz, J = 2.4 Hz, 1H), 6.66 (d, J = 7.6 Hz, 1H), 4.64 (s, 2H), 4.58 (s, 2H), 3.33–3.27 (m, 2H), 3.26–3.21 (m, 2H), 3.19–3.13 (m, 4H). ^13^C NMR (150 MHz, DMSO-*d_6_*): δ 168.0, 167.0, 157.6, 157.6, 154.1, 148.4, 147.4, 138.7, 137.8, 130.0, 119.1, 114.5, 99.9, 97.1, 67.6, 50.3, 40.3, 40.1, 38.0, 37.4. HRMS (ESI) *m*/*z*: calcd for C_20_H_28_N_11_O_4_^+^ [M+H]^+^: 486.2320; found 486.2319.

Ditrifluoroacetate salt of N-(2-guanidinoethyl)-2-(5-(2-((2-guanidinoethyl)amino)-2-oxoethoxy)-11-oxonaphtho[2′,1′:4,5]imidazo[1,2-c]pyrimidin-10(11H)-yl)acetamide **7b**

Starting from **5b**, the title compound was obtained with a yield of 50% as a yellowish amorphous solid. HPLC rt 6.5 min. ^1^H NMR (600 MHz, DMSO-*d_6_*): δ 9.43 (br s, 2H), 8.84 (br s, 1H), 8.58–8.47 (m, 3H), 8.05–7.76 (br s, 4H), 7.88 (s, 1H), 7.75 (dd, J = 8.0 Hz, J = 7.0 Hz, 1H), 7.65 (dd, J = 8.4 Hz, J = 7.0 Hz, 1H), 7.62 (d, J = 7.6 Hz, 1H), 6.82 (d, J = 7.6 Hz, 1H), 4.79 (s, 2H), 4.69 (s, 2H), 3.39–3.36 (m, 2H), 3.28–3.23 (m, 2H), 3.20 (t, J = 5.8 Hz, 2H), 3.19–3.16 (m, 2H). ^13^C NMR (150 MHz, DMSO-*d_6_*): δ 167.7, 167.0, 157.7, 150.1, 147.5, 146.9, 136.4, 134.4, 127.3, 125.5, 125.5, 125.4, 123.6, 123.1, 121.9, 97.5, 94.8, 67.7, 50.5, 40.4, 40.1, 38.0, 37.4. HRMS (ESI) *m*/*z*: calcd for C_24_H_30_N_11_O_4_^+^ [M+H]^+^: 536.2477; found 536.2475.

#### 2.1.8. Dihydrochloride Salt of N-(2-Aminoethyl)-2-(5-(2-((2-aminoethyl)amino)-2-oxoethoxy)-11-oxonaphtho[2′,1′:4,5]imidazo[1,2-c]pyrimidin-10(11H)-yl)acetamide (**5b**·2HCl)

To a suspension of **5b** (90 mg, 0.2 mmol) in CH_3_OH (5 mL), 7N aqueous HCl solution (0.2 mL) was added, and the resulting mixture was stirred at room temperature overnight. Then, Et_2_O (50 mL) was added dropwise with vigorous stirring, and the formed precipitate was filtered, washed with Et_2_O (2 × 25 mL), and dried in vacuo, yielding **5b**·2HCl as a light beige crystalline solid (94%). M.p. ~165 °C with decomposition. ^1^H NMR (600 MHz, D_2_O): δ 8.13 (d, J = 8.2 Hz, 1H), 7.96 (d, J = 8.1 Hz, 1H), 7.65 (t, J = 7.4 Hz, 1H), 7.59 (d, J = 7.6 Hz, 1H), 7.58 (t, J = 7.1 Hz, 1H), 7.01 (s, 1H), 6.74 (d, J = 7.6 Hz, 1H), 4.80 (s, 2H, overlaps with DHO), 4.60 (s, 2H), 3.74 (t, J = 6.2 Hz, 2H), 3.70 (t, J = 5.9 Hz, 2H), 3.31 (t, J = 6.2 Hz, 2H), 3.30 (t, J = 5.9 Hz, 2H). ^13^C NMR (150 MHz, D_2_O): δ 171.3, 169.3, 150.3, 146.8, 145.8, 138.6, 128.7, 128.0, 126.5, 124.1, 123.2, 122.4, 122.4, 121.1, 96.0, 93.3, 67.0, 51.4, 39.1, 39.0, 37.1, 36.8.

### 2.2. Cell-Based Assays

#### 2.2.1. Cell Lines

Cell lines MCF7′, VA13, A549, and HEK293T were maintained in DMEM/F-12 (Thermo Fisher Scientific, Madison, WI, USA) culture medium containing 10% fetal bovine serum (Thermo Fisher Scientific, Madison, WI, USA), 50 µg/mL penicillin, and 0.05 mg/mL streptomycin at 37 °C (Thermo Fisher Scientific, Madison, WI, USA) in 5% CO_2_. Cells were maintained at 37 °C in a MCO-18AC humidified incubator (Sanyo, Tokyo, Japan) supplied with 5% CO_2_. The cell cultures were tested for the absence of mycoplasma and validated by STR.

#### 2.2.2. MTT Assay

The cytotoxicity of the substances was tested using the MTT (3-(4,5-dimethylthiazol-2-yl)2,5-diphenyl tetrazolium bromide) Mosmann assay [23] with some modifications. A total of 2500 cells per well for the MCF7′, HEK293T, and A549 cell lines or 4000 cells per well for the VA13 cell line were plated out in 135 µL of DMEM-F12 medium (Gibco, USA) in a 96-well plate and incubated in a 5% CO_2_ incubator for 20 h without treatment. Then, 15.8 µL of the corresponding medium-DMSO solutions of the substances being tested was added to the cells in triplicate for each dilution (eight concentrations with three-times dilutions with final concentrations in cells ranging from 0.05 to 100 µM; the final DMSO concentrations in the medium were 0.5% or less) and incubated for 72 h. Doxorubicin was used as a control substance. Then, the MTT reagent (Paneco LLC, Moscow, Russia) was added to the cells up to the final concentration of 0.5 g/L (10× stock solution in PBS was used) and incubated for 1.5 h (MCF7′, HEK293T and A549 cells) or 3 h (VA13 cells) at 37 °C in the 5% CO_2_ incubator. Then, the MTT solution was discarded, and 140 µL of DMSO (PharmaMed LLC, Moscow, Russia) was added. The plates were agitated on a shaker (120 rpm) to dissolve the formazan. Absorbance was measured using a Victor X5 microplate reader (PerkinElmer, Waltham, MA USA) at a wavelength of 555 nm. The results were used to construct dose–response curves by non-linear regression approximation of the normalized data and to estimate IC_50Abs_ values (IC_50Abs_ is the concentration resulting in two-fold decrease in the number of cells compared to untreated cells) with GraphPad Prism 5 (GraphPad Software Inc., La Jolla, CA, USA).

#### 2.2.3. Apoptosis/Necrosis Assay

A total of 3 × 10^5^ cells per well of the A549 cell line were seeded in 2 mL of DMEM-F12 medium (Gibco, USA) in 6-well plates, and then incubated in the 5% CO_2_ incubator for 20 h without treatment. After that, the medium-DMSO solutions of the compounds under test in amounts of 1–33 µL were added (the DMSO concentrations in the wells were 1.5% or less) and the cells were incubated for 24 h. The cells were collected (a sedimentation procedure was performed at 200–300 g) in 1.5 mL tubes, flushed with PBS, and resuspended in 1X Annexin V binding buffer consisting of 0.01 M HEPES (pH 7.4), 0.14 M NaCl, and 2.5 mM CaCl_2_ solution. Then, 5 µL of Annexin V–FITC (A13201, ThermoFisher, Waltham, MA, USA) was added per 100 µL of cell solution in a 1× binding buffer, and the cells were incubated for 15 min at room temperature. Afterwards, the cells were sedimented, and the solution was replaced with 1X Annexin V binding buffer containing propidium iodide (2.5 µL of PI (1 mg/mL, ThermoFisher, USA) per 100 µL of buffer), incubated for 15 min at room temperature. Then, the cells were analyzed with a LongCyte C3090 flow cytometer (Challenbio, Beijing, China) after staining for 2 h.

Caspase 3/7 activation was analyzed with the Muse^®^ Caspase-3/7 Kit (Cytek Biosciences, Fremont, CA, USA). The cells were prepared in the same way as in the previous assay, but they were resuspended in 500 µL of growth medium, followed by staining and analysis of 100 µL of the suspension in accordance to the manufacturer’s protocol.

#### 2.2.4. Cell Cycle Assay

A total of 1 × 10^5^ cells per well of the A549 cell line were seeded in 1 mL of DMEM-F12 medium (Gibco, USA) in a 12-well plate and incubated in the 5% CO_2_ incubator for 20 h without treatment. Then, 2 µL of medium-DMSO solutions of the substances under test (the final DMSO concentrations in the medium were 0.5% or less) was added to the cells and incubated for 24 h. The cells were collected (a sedimentation procedure was performed at 200–300 g) in 1.5 mL tubes, flushed with PBS, and stained with the staining solution (500 µL, RPMI1640 medium (Gibco, USA), Hoechst-33342 (10 µg/mL, Invitrogen, Waltham, MA, USA) with TritonX100 (0.1%) and HEPES-KOH (10 mM pH 7.3)) for 15 min at room temperature. The cells were analyzed with a LongCyte C3090 (Challenbio, Beijing, China) or Amnis FlowSight (Luminex, Austin, TX, USA) flow cytometer after staining for 0.5 h.

### 2.3. Oligonucleotide-Based Assays

#### 2.3.1. FRET-Melting Assay

Oligodeoxyribonucleotides (ODNs) labeled with 6-carboxyfluorescein (FAM) and Black Hole Quencher 1 (BHQ1) at 5′- and 3′-termini, respectively, and unlabeled oligodeoxynucleotide ds26 were purchased from Litekh (Moscow, Russia) (purity > 95%, HPLC). The ODN sequences are presented in Table S1. Solutions of the ODNs (1 μM) were prepared in 20 mM sodium phosphate buffer, pH 7.4, supplemented with 10 mM KCl (buffer 1), in all cases except VEGF, for which a 5 mM sodium phosphate buffer, pH 7.4, supplemented with 25 mM LiCl (buffer 2) was used. The compounds under test were added to ODN solutions to final concentrations of 1–20 μM (1:1, 1:2, 1:5, 1:10, or 1:20 ODN:compound ratio). The mixtures were heated to 95 °C for 5 min and then cooled on ice to facilitate the intra-molecular folding of ODN structures. FRET melting curves were obtained using a QuantStudio 5 PCR system (Thermo Fisher Scientific, USA). Changes in fluorescence at 520 nm were recorded every 0.3 °C during stepwise heating of the samples at an average rate of 1.5 °C/min. Melting temperatures were determined from the maxima of first derivatives of the melting curves. For selectivity analysis, labeled G4-forming oligonucleotides were mixed with pre-annealed unlabeled ds26 (G4 and ds26 concentrations: 0.5 μM and 10 μM, respectively); then, the compound under test was added to a final concentration of 10 μM, and the melting experiments were performed as described above.

#### 2.3.2. MST Assay

ODNs labeled with hexachlorofluorescein (HEX) at the 5′-terminus were purchased from Litekh (Russia) (purity > 95%, HPLC). The ODN sequences are presented in Table S2. The pre-annealed 100 nM solution of 5′-HEX-labeled cKit1 ODN in buffer 1, STAT ODN in buffer 1, or VEGF ODN in buffer 2 was mixed 1:1 (*v*/*v*) with two-fold dilutions of the compound under test in the corresponding buffer supplemented with 5% DMSO and 0.5% Tween 20. The final concentrations of the compounds being tested ranged from 0 to 240 μM. The mixtures were incubated at room temperature for 10 min prior to measurements. Microscale thermophoresis (MST) curves were recorded using a Monolith NT.115 instrument and standard capillaries (NanoTemper, Germany) at 22 °C in GREEN mode. To calculate *K_d_* values, the dependence of MST data on the concentration of the compound under test was analyzed using MO. Affinity Analysis software (NanoTemper, Germany).

### 2.4. Dual Luciferase Reporter (DLR) Assay

The HEK293 cells were cultured in a 25-cm^2^ flask until 70–80% confluency was achieved. Once the required density was reached, the cells were transfected with the pC-Kit1 plasmid (Addgene plasmid #118983) using the GenJect-40 reagent (Molecta). After transfection for 4 h, the cells were plated into a 96-well plate at a seeding density of 4 × 10^4^ cells/well. Immediately after seeding, various concentrations of **5b** (5, 20, and 50 μM) were added to the cells. Luciferase activity was measured 24 h after the treatment using the Dual-Glo^®^ Luciferase Assay System (Promega, Madison, WI, USA). All assays were performed in triplicate.

### 2.5. Fluorescent Intercalator Displacement (FID) Assay

An Ethidium bromide (EtBr) displacement assay was performed in a 40 µL volume in 384-well black, flat-bottom, polystyrene microtiter plates. Solutions of EtBr in Tris buffer (0.05 M Tris·Cl, pH 7.5, 0.1 M KCl) at a concentration of 6 µM, 0.016 g/L calf thymus DNA that corresponds to 24 µM of nucleotide pairs and the compound with final concentrations of 4, 16, 62.5, and 250 µM dissolved in DMSO (final concentration up to 3%) were prepared. The compound and blank solutions contained the same percentage of DMSO. Once the ethidium bromide solution had been added, the plates were protected from light. The mixtures were incubated for 15 min, and EtBr fluorescence (545 nm for excitation and 595 nm for emission) was measured using Victor X5 (PerkinElmer).

## 3. Results and Discussion

### 3.1. Synthesis of the Compounds

**Scheme 1:**
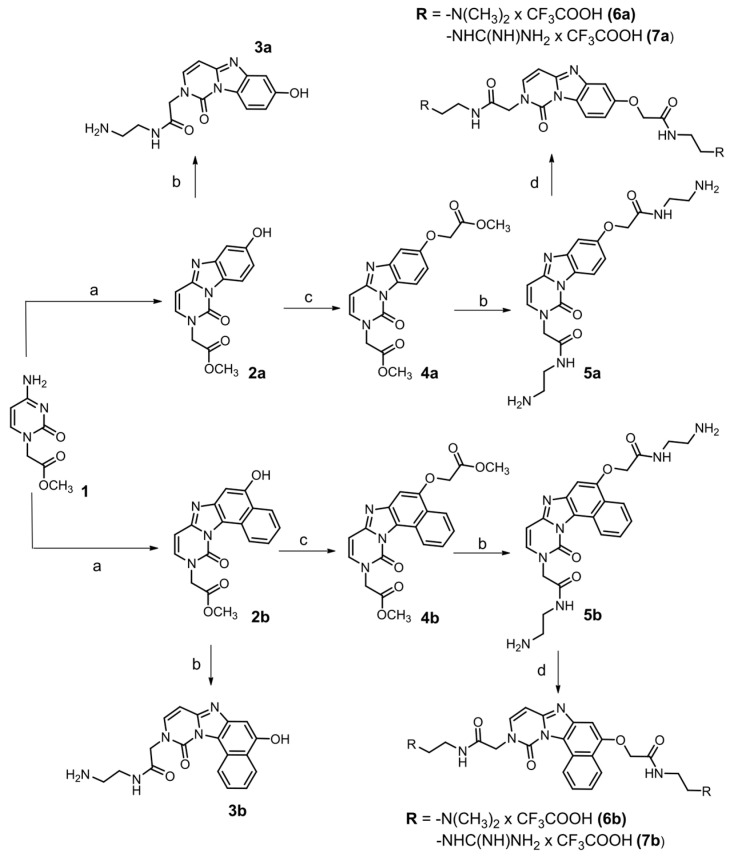
Synthesis of benzo- and naphtho-imidazopyrimidinone derivatives. Reagents and conditions: (a) 1,4-benzoquinone, 0.1 M sodium acetate buffer (pH 4.5), 37 °C, 24 h for **2a** (60%) or 1,4-naphthoquinone, 0.1 M sodium acetate buffer (pH 4.5)/C_2_H_5_OH (2:1, *v*:*v*), 50 °C, 7d for **2b** (21%); (b) ethane-1,2-diamine, CH_3_OH, 50°, 48 h for **3a** (83%), **3b** (80%), **5a** (91%), **5b** (90%); (c) DBU, methyl 2-bromoacetate, CH_2_Cl_2_, rt, 3h for **4a** (43%) and **4b** (48%); (d) CH_2_O, NaCNBH_3_, AcOH, MeOH, 0 °C→rt, 2h for **6a** (38%) and **6b** (33%) and 1H-pyrazole-1-carboxamidine hydrochloride, DIPEA, DMSO, 60 °C 3h for **7a** (47%) and **7b** (50%).

To construct tri- and tetracyclic heteroaromatic systems, we used a reported reaction of 2′-deoxycytidine with 1,4-benzoquinone in sodium acetate buffer (pH 4.5) [19], but started from N1-substituted cytosine **1** (Scheme 1). The treatment of **1** with 1,4-benzoquinone afforded tricyclic derivative **2a**. For the preparation of tetracyclic derivative **2b**, 1,4-naphthoquinone was used with an addition of C_2_H_5_OH to improve its solubility, and the reaction time was prolonged. Then, **2a**–**b** were reacted with ethane-1,2-diamine in CH_3_OH at 50 °C, affording derivatives **3a**–**b** with an amino-containing tether. The alkylation of the hydroxyl group in the heteroaromatic moiety of **2a**–**b** was carried out by the addition of 1,8-diazabicyclo[5.4.0]undec-7-ene (DBU), followed by methyl 2-bromoacetate in CH_2_Cl_2_, affording intermediates **4a**–**b**. The subsequent substitution of the methoxy groups in esters **4a**–**b** with ethane-1,2-diamine in CH_3_OH at 50 °C led to ligands **5a**–**b** bearing two amino-containing tethers. The derivatives **6a**–**b** were prepared via the reductive methylation of **5a**–**b** [24]**.** To convert the amino groups of **5a**–**b** into the guanidino ones of **7a**–**b**, a standard guanidinylating agent, 1H-pyrazole-1-carboxamidine hydrochloride, was used [25,26].

### 3.2. Cytotoxicity Assays

**Figure 2:**
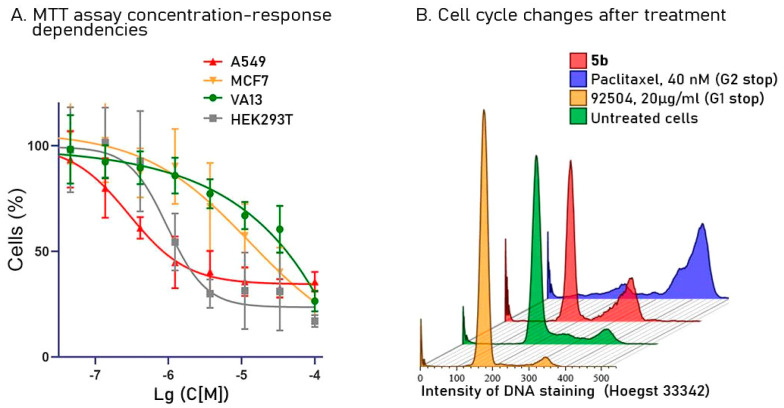
(**A**) Dose–response dependencies of cytotoxicity for HEK293T, MCF7′, A549, and VA13 cell lines after a 72 h treatment with **5b**. (**B**) Changes in cell cycle distribution of lung cancer cells A549 after a 20 h treatment with **5b**, paclitaxel, and 92504.

The cytotoxicity of benzo- and naphtho-imidazopyrimidinyl derivatives and their intermediates was evaluated via an MTT assay [21], using human breast cancer cell line MCF7′ (fast-growth subclone), human lung epithelial carcinoma cell line A549, and non-cancerous lung fibroblast cell line VA13 (Table 1). The cytotoxicity for immortalized human embryonic kidney cell line HEK293T as control non-cancerous fast-growth cells was also studied. Most of the compounds were non- or weakly cytotoxic both for cancerous and control cell lines. The introduction of an additional benzene ring into the heteroaromatic scaffold appears to determine the activity throughout the naphtho series **b**. However, only **5b** with two amino-bearing tethers demonstrated significant cytotoxicity, with the selectivity index (SI) being 3.0 and 17.3 for the VA13/MCF7 and VA13/A549 pairs, respectively (Table 1, Figure 2A). Its congeners carrying dimethylamino and guanidino moieties exhibited weak activity. For some heterocyclic scaffolds, the lower activity of dimethylamino- and guanidino-substituted compounds compared to derivatives with amino groups has been reported [27,28,29]. Of note, **5b** outperformed commonly used chemotherapy drugs, such as cisplatin, doxorubicin, and 5F-uracil [30,31], in terms of selectivity.

The treatment with **5b** did not cause complete cell death even at high concentrations (Figure 2A), and apoptosis induction is not the main mechanism of action of the compound (Table S3, Figures S1 and S2). We checked if **5b** had cytostatic activity and found that it caused G2 arrest in A549 cells (Table 2, Figure 2B). This effect was significant, though less prominent than after the treatment of cells with the well-established cytostatic drug paclitaxel with G2-arresting activity [36,37].

### 3.3. Verification of DNA Targets: FRET-Melting and Microscale Thermophoresis (MST) Assays

In an attempt to elucidate the mechanism of action underlying the anti-proliferative properties, we investigated the ability of the most active tetracyclic derivatives to interact with a panel of biologically relevant DNA secondary structures. The non-active tricyclic analogs were used as negative controls. Compound 5b contains a planar condensed aromatic core and positively charged tethers, allowing for interactions with the DNA duplex leading to DNA damage. In addition, carbazole-based derivatives can stabilize G4s that regulate oncogene transcription and inhibit telomere elongation [38]. Telomeric and c-Myc G4s have previously been reported as the main targets of carbazole-based G4 ligands [3].

Here, we used a FRET-melting assay with FAM/BHQ-labeled ODNs to evaluate the stabilizing effects of the synthesized compounds on the DNA duplex and G4s. The 23-mer hairpin Hair was chosen as a model duplex [39]. In addition to telomeric 22AG [40] and c-Myc [41] G4s, three G4s from the promoters of oncogenes cKit1 [42], STAT [43], and VEGF [44], as well as three imperfect G4s (Ct1, BclT, and 22CTA) [45,46], were also included. To verify a topological preference, if any, the G4 set encompassed various topologies: antiparallel (22CTA and STAT), parallel (cMyc, cKit1, and VEGF), hybrid (22AG) and mixed hybrid/antiparallel (Ct1 and BclT) G4s. Furthermore, three of them, namely, cKit1, STAT, and VEGF, contained long loops that can be involved in the interactions with G4 stabilizers. In order to determine the effective concentration range and roughly evaluate the binding affinity of the compounds to G4 targets, the concentration dependence of the stabilizing effects was analyzed in a series of titration assays (Tables S4 and S5).

**Figure 3:**
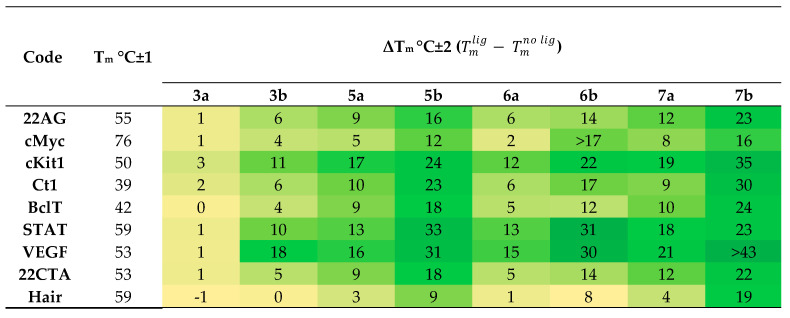
Heatmap of the influence of the compounds on the thermal stability of DNA targets. The concentrations of the compounds and the targets were 1 and 20 µM, respectively. For all targets except VEGF, buffer 1 (20 mM sodium phosphate, pH 7.4, 10 mM KCl) was used. For VEGF, buffer 2 (5 mM sodium phosphate, pH 7.4, 25 mM LiCl) was used.

The G4/hairpin-stabilizing effects of all compounds are summarized in Figure 3. Both tricyclic and tetracyclic compounds increased the T_m_ of G4s, but the stabilization of the hairpin was observed only in the naphtho series b. The compounds demonstrated no apparent selectivity for particular types of G4 topologies. The smallest stabilizing effect was observed for c-Myc G4, which has the highest intrinsic thermal stability (the highest T_m_ value in the absence of the ligand), in all cases except for the case of 6b. Weak to moderate stabilizing effects were predominantly observed for telomeric (22AG) and imperfect (BclT, 22CTA, and Ct1) G4s, except for the highly efficient stabilizers 5b and 7b. Both tri- and tetracyclic derivatives gave the highest increase in melting temperature for cKit1, STAT, and VEGF G4s. Taking into account that the last three G4s have the longest loops, the efficacy of G4 stabilization could depend on loop–ligand interactions.

Tricyclic derivative 3a with a positively charged amino-containing tether demonstrated no stabilizing effect. Among the di-substituted tricyclic derivatives, guanidylated 7a exhibited the highest stabilizing effect, while 6a with dimethylamino-bearing arms showed the lowest one. A similar dependence of the effect on the type and number of tethered groups (3b < 5b < 6b < 7b in most cases) was observed among the tetracyclic compounds.

The introduction of an additional benzene ring into the benzoimidazopyrimidinone scaffold had a positive impact on the stabilization of the G4s and the hairpin, presumably due to additional stacking contacts with outer G tetrads or canonical Watson–Crick base pairs. Mono-substituted compounds were the weakest stabilizers in both series, suggesting that the number of positively charged terminal groups influences stabilization efficacy, presumably due to electrostatic interactions with the sugar-phosphate backbone. The superiority of the guanidylated derivatives can be explained by the high pK_a_ value of the guanidino group [47]. The lower efficacy of N-dimethylated derivatives can result from charge shielding and steric hindrance [27].

In summary, the tetracyclic molecules exhibited a more pronounced stabilization of both G4s and duplex DNA, that is mostly consistent with their anti-proliferative activity. In contrast, the tricyclic derivatives that were inactive in MTT assays demonstrated lower G4-stabilizing effects and no impact on duplex thermal stability.

**Figure 4:**
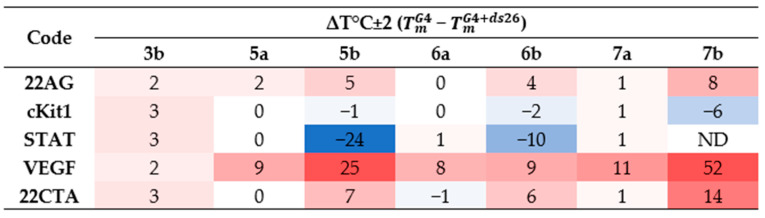
Heatmap of changes in the G4-stabilizing effects of the compounds in the presence of duplex DNA. ND—not determined. The concentrations of the compounds, ds26, and the targets were 10 µM, 10 µM, and 0.5 µM, respectively. Buffer 1 (20 mM sodium phosphate, pH 7.4, 10 mM KCl) was used for all targets, and buffer 2 (5 mM sodium phosphate, pH 7.4, 25 mM LiCl) was used for VEGF..

To assess G4 vs. duplex selectivity, we chose five G4 targets that were stabilized most effectively in the previous assay, and performed FRET melting in the presence of derivatives and a competitor—unlabeled hairpin ds26 at a 20-fold excess relative to G4 (Figure 4). The mono-substituted derivative 3a was excluded due to the lack of effect. For all investigated compounds, except for the mono-substituted tetracyclic compound 3b, the melting point of VEGF decreased significantly in the presence of ds26. Regarding tricyclic molecules, the stabilizing effect on 22AG, 22CTA, STAT, and cKit1 G4s remained almost unchanged, with the derivatives demonstrating a preference for these G4 targets over duplex DNA. The stabilizing effects of di-substituted tetracyclic compounds mainly decreased in the presence of the competitor. Surprisingly, a high stabilizing effect was observed for STAT in the presence of ds26, which might be explained by the formation of a triple G4/ds26/derivative complex. cKit1 G4 can be highlighted as a preferred target for tetracyclic derivatives, but, upon entering the cells, they can target both telomeric G4 and duplex DNA due to their high abundance.

**Figure 5:**
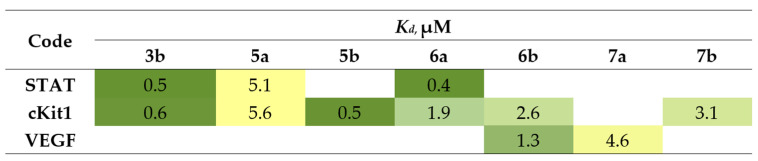
Heatmap of the dissociation constants *K_d_* of G4:derivative complexes according to MST. ND—not determined.

For the most promising compounds, the dissociation constants (*K_d_*) of their complexes with proto-oncogenic G4s STAT, VEGF, and cKit1 were determined using microscale thermophoresis (MST) assays with 5′-HEX-labeled G4 ODNs. The results are presented in Figure 5. The *K_d_* values of 3b/cKit, 5b/cKit, 3b/STAT, and 6a/STAT were in the submicromolar range, and the rest of the complexes tested showed micromolar *K_d_*. Despite causing only a moderate increase in G4 T_m_, 3b turned out to be the most efficient G4 binder.

In order to assess the impact of 5b on cKit expression, a DLR assay with the pC-Kit1 plasmid (Addgene plasmid #118983) that contains two luciferase genes (Renilla/Firefly) was performed. The expression of Renilla is controlled by the c-Kit promoter containing the cKit1 G4-forming sequence, while the expression of Firefly is under control by a non-G4-forming promoter sequence (the HSV TK promoter). After transfection of the plasmid into HEK293T cells for 4 h, followed by a 24 h treatment with 5b, no significant changes in Renilla/Firefly expression ratio compared with the control were observed (Figure S3), suggesting that G4 in the cKit promoter region is not the main target of 5b.

According to the FRET-melting assay, there is evidence for interactions of 5b with DNA structures. The FID assay demonstrated that 5b is a DNA intercalator, though an order of magnitude weaker one than EtBr (Figure S4).

The problem with DNA-targeting compounds is adverse effects and common toxicity in combination with a low selectivity of action. The aims of this study were to evaluate the prospects for further study of this class of derivatives; and to determine whether it is necessary to synthesize additional compounds of this class, study their detailed molecular mechanism, and conduct comprehensive studies in animal models. To evaluate the prospects of 5b for further application in vivo, we prepared its water-soluble dihydrochloride salt 5b·2HCl (see Section 2.1. Chemical synthesis) and performed tolerance tests on mice. Compound 5b·2HCl up to 20 mg/kg did not demonstrate acute toxic effects and caused no changes in mice behavior or decrease in body weight after a 72 h treatment. The results support the prospects of further search for the cellular targets and evaluation in vivo. Hopefully, the unraveling of the molecular target of 5b will be accomplished in the future.

## 4. Conclusions

Novel derivatives of benzo[4,5]- and naphtho[2′,1′:4,5]imidazo[1,2-c]pyrimidinones were synthesized and evaluated as anti-proliferative agents. Tetracyclic compound 5b with two amino-containing tethers inhibited metabolic activity in the low-micromolar range, with notable selectivity for cancerous and fast-growing cells; however, it did not cause complete cell death. In attempt to explore its mechanism of action, the biological properties of 5b were further studied. A cell cycle assay demonstrated that 5b causes G2 arrest without inducing apoptosis. The interaction of the compounds with DNA targets was of particular interest, and FRET-melting and MST experiments revealed that both benzo[4,5]- and naphtho[2′,1′:4,5]imidazo[1,2-c]pyrimidinones can bind and stabilize G4s, but only the latter can interact with the DNA duplex. A dual luciferase reporter assay showed that 5b does not regulate gene expression under control by the promoter harboring the cKit1 G4-forming sequence. In contrast, an FID assay revealed the intercalating properties of 5b that could be partly responsible for its anti-proliferative activity. Finally, a water-soluble dihydrochloride salt of 5b up to 20 mg/kg was shown not to be toxic for mice after a 72 h treatment. Here, we have demonstrated the first evidence that naphtho[2′,1′:4,5]imidazo[1,2-c]pyrimidinone derivative is a DNA-targeting agent that causes cell cycle halting and possesses selectivity for cancer cell lines. Thus, the lead compound looks promising for further investigation; however, the discovery of its molecular targets is necessary.

## Figures and Tables

**Table 1:** Anti-proliferative effects of benzo- and naphtho-imidazopyrimidinone derivatives against HEK293T, MCF7′, A549, and VA13 cell lines, and selectivity indexes after 72 h incubation and viability measurement using the Mosmann assay (MTT). VA13 and HEK293T are slow- and fast-growth non-cancerous cell lines, respectively; MCF7′ and A549 cell lines are of breast and lung origin, respectively.

Compound	IC_50abs_, µM				SI	
	HEK293T	MCF7′	A549	VA13	VA13/MCF7	VA13/A549
**2a**	>100	>100	>100	~100	ND	ND
**3a**	>100	>100	>100	>100	ND	ND
**4a**	>100	>100	>100	>100	ND	ND
**5a**	~100	>100	>100	>100	ND	ND
**6a**	>100	>100	>100	>100	ND	ND
**7a**	~100	>100	>100	>100	ND	ND
**2b**	6.1 ± 0.3	30 ± 1	34 ± 2	60 ± 6	2.0	1.7
**3b**	32 ± 1	95 ± 3	94 ± 5	73 ± 2	0.8	0.8
**4b**	44 ± 1.9	~100	~100	~100	ND	ND
**5b**	2.6 ± 0.2	20 ± 2	3.6 ± 0.5	62 ± 5	**3.0**	**17.3**
**6b**	~100	>100	>100	>100	ND	ND
**7b**	41 ± 4	~100	76 ± 6	~100	ND	ND
Dox	0.02 ± 0.01 ^2^	0.04 ± 0.02 ^2^	0.014 ± 0.005 ^2^	0.11 ± 0.04 ^2^	3.0	7.9
5F-Uracil	ND	10.0 ± 0.2 ^3^	4 ± 1 ^1^	13.6 ± 0.6 ^1^	1.4	3.4
Cisplatin	3.4 ± 1.7 ^4^	5.75 ± 0.02 ^5^	2.69 ± 0.05 ^1^	2.04 ± 0.08 ^1^	0.4	0.8

ND—not determined ^1–5^ Data about cytotoxicity of known drugs were retrieved from ^1^ [31], ^2^ [32], ^3^ [33], ^4^ [34], and ^5^ [35].

**Table 2:** Cell cycle effects of **5b** and G1 and G2 phase-arresting drugs 92504 and paclitaxel, respectively.

Sample	G1, %	S, %	G2, %
**5b**	58	6	36
92504 (cause G1-stop)	91	3	6
Paclitaxel (cause G2-stop)	10	8	82
Untreated cells	72	17	11

## Data Availability

Data are contained within the article and supplementary materials.

## Supplementary Materials

The following supporting information can be downloaded at: https://www.mdpi.com/article/10.3390/biom13111669/s1, Figure S1: Determination of the mode of cell death using Annexin-V and PI staining and flow cytometric analysis; Figure S2: Determination of the mode of cell death using caspase 3/7 staining and flow cytometric analysis; Figure S3: The dual luciferase reporter assay using the pC-Kit1 plasmid; Figure S4: The EtBr displacement assay data for 5b (4, 16, 62.5, 250 µM) demonstrates intercalation into DNA helix, Acridin-9-amine was used as a control sample; Table S1: Sequences of the ODNs used in FRET-melting assays; Table S2: Sequences of the ODNs used in the MST study; Table S3: Determination of the mode of cell death by 5b using Annexin-V and PI staining and flow cytometric analysis; Table S4: Titration of DNA-targets with the benzo-imidazopyrimidinone derivatives; Table S5: Titration of DNA-targets with the naphtho-imidazopyrimidinone derivatives.
