# Supplementary material for: Synthesis and Biological Evaluation of Benzo [4,5]- and Naphtho[2′,1′:4,5]imidazo[1,2-c]pyrimidinone Derivatives
Source: Biomolecules. 2023 Nov 20;13(11):1669. doi: 10.3390/biom13111669 (PMC10669118; doi:10.3390/biom13111669)
Supplement: Supplementary file 1 [file biomolecules-13-01669-s001.zip › biomolecules-2693582-supplementary.pdf]

Supplementary

Synthesis and biological evaluation of benzo[4,5]- and naphtho[2',1':4,5]imidazo[1,2-c]pyrimidinone derivatives

Polina Kamzeeva[1,†], Nikolai Dagaev [2,†], Sofia Lizunova[3], Yuri Khodarovich[1,4], Anna Sogomonyan[1], Anastasia Kolchanova[2], Vadim Pokrovski[5,6], Vera Alferova[1], Alexey Chistov[1], Artur Eshtukov-Shcheglov[1], Elizaveta Eshtukova-Shcheglova[7], Evgeny Belyaev[8], Dmitry Skvortsov[2,*], Anna Varizhuk[3,9,10] and Andrey Aralov[1,3,10,*]

[1] Shemyakin-Ovchinnikov Institute of Bioorganic Chemistry, Russian Academy of Sciences, 117997 Moscow, Russia; polinabast@yandex.ru (P.K.)

[2] Department of Chemistry, Faculty of Bioengineering and Bioinformatics, Lomonosov Moscow State University, 119991 Moscow, Russia; nikolas.dagaev@yandex.ru (N.D.)

[3] Lopukhin Federal Research and Clinical Center of Physical-Chemical Medicine of Federal Medical Biological Agency, 119435 Moscow, Russia

[4] Research and Educational Resource Center for Cellular Technologies, The Peoples' Friendship University of Russia, 117198 Moscow, Russia

[5] N.N. Blokhin Cancer Research Center, 115478 Moscow, Russia

[6] Research Institute of Molecular and Cellular Medicine, RUDN University, 117198 Moscow, Russia

[7] Lomonosov Institute of Fine Chemical Technology, MIREA-Russian Technological University, 119571 Moscow, Russia

[8] Frumkin Institute of Physical Chemistry and Electrochemistry, Russian Academy of Sciences, 119071 Moscow, Russia

[9] Moscow Institute of Physics and Technology, 141701 Dolgoprudny, Russia

[10] G4_Interact, USERN, University of Pavia, 27100 Pavia, Italy

* Correspondence: skvorratd@mail.ru (D.S.), Baruh238@mail.ru (A.A.)

†These authors contributed equally to this work.

Table S1. Sequences of the ODNs used in FRET-melting assays.

Code	Sequence, 5'→3'
**22AG**	FAM-AGGGTTAGGGTTAGGGTTAGGG-BHQ1
**cMyc**	FAM-TGAGGGTGGGTAGGGTGGGTAA-BHQ1
**cKit1**	FAM-GGGAGGGCGCTGGGAGGAGGG-BHQ1
**Ct1**	FAM-GGTGACAGGGGTATGGGGAGGGG-BHQ1
**BclT**	FAM-GGGGGCCGTGGGGTGTGAGCTGGGG-BHQ1
**STAT**	FAM-GGGCTGGGGATGGGGAGGGGG-BHQ1
**VEGF**	FAM-GGGAGGGTTGGGGTGGG-BHQ1
**22CTA**	FAM-AGGGCTAGGGCTAGGGCTAGGG-BHQ1
**Hair**	FAM-GTATAGCTATTTTTATAGCTATA-BHQ1
**ds26**	CAATCGGATCGAATTCGATCCGATTG

Table S2. Sequences of the ODNs used in the MST study.

Code	Sequence, 5'→3'
**cKit1**	HEX-GGGAGGGCGCTGGGAGGAGGG
**STAT**	HEX-GGGCTGGGGATGGGGAGGGGG
**VEGF**	HEX-GGGAGGGTTGGGGTGGG

Table S3. Determination of the mode of cell death by **5b** using Annexin-V and PI staining and flow cytometric analysis.

Sample	Q1 (necrosis), %	Q2 (necrosis / late apoptosis), %	Q3 (apoptosis), %	Q4 (viable), %
**5b** (28 µM)	4.95	2.55	0.96	91.5
Doxorubicin (9.2 µM)	8.47	7.85	14.5	69.2
Control	7.85	6.03	1.25	84.9

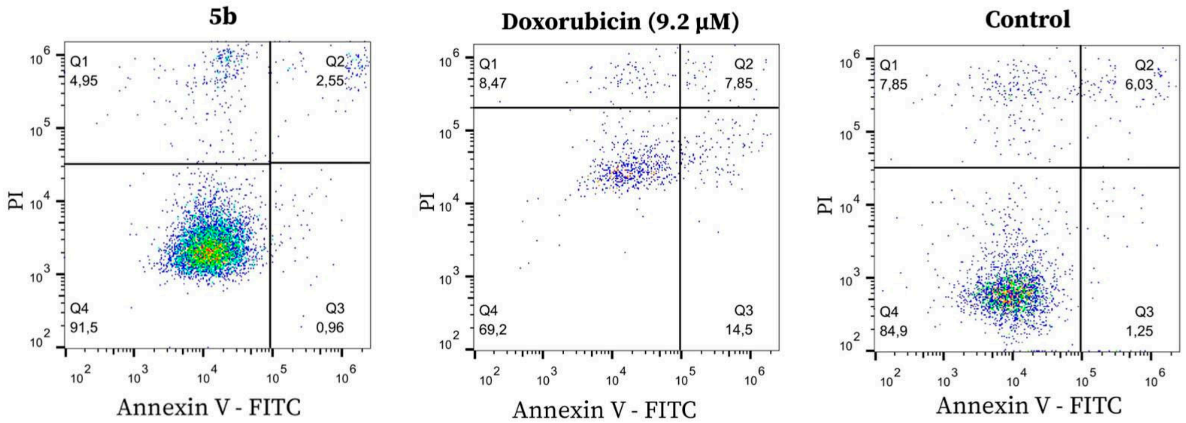

Figure S1. Determination of the mode of cell death using Annexin-V and PI staining and flow cytometric analysis. Representative biparametric histogram obtained after 24 h treatment of A549 cells with **5b** (28 µM), doxorubicin (9.2 µM) and without treatment (control).

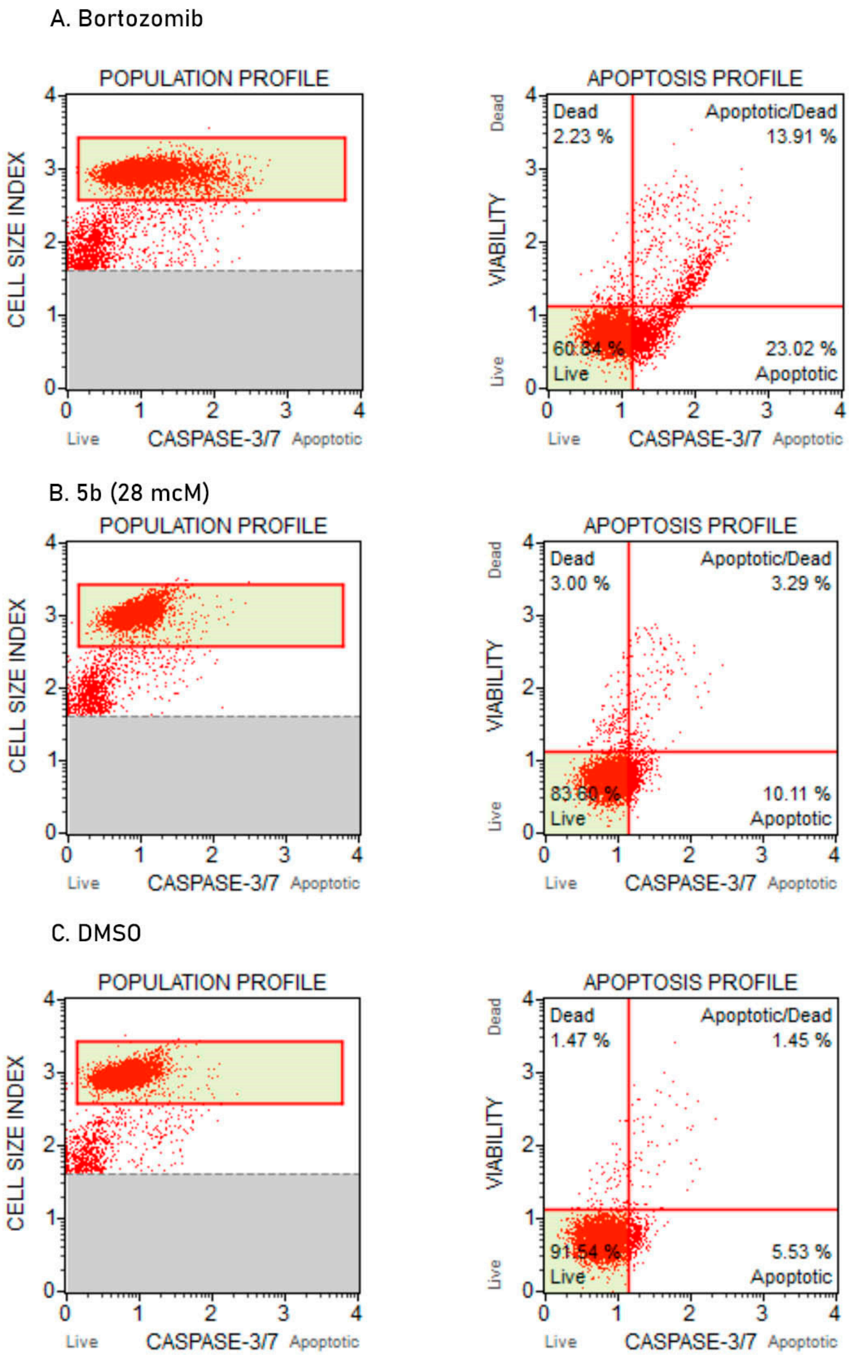

Figure S2. Determination of the mode of cell death using caspase 3/7 staining and flow cytometric analysis. Biparametric histograms obtained after 24 h treatment of A549 cells with 5b (28 μM), bortezomib (10 μM) and without compound (DMSO).

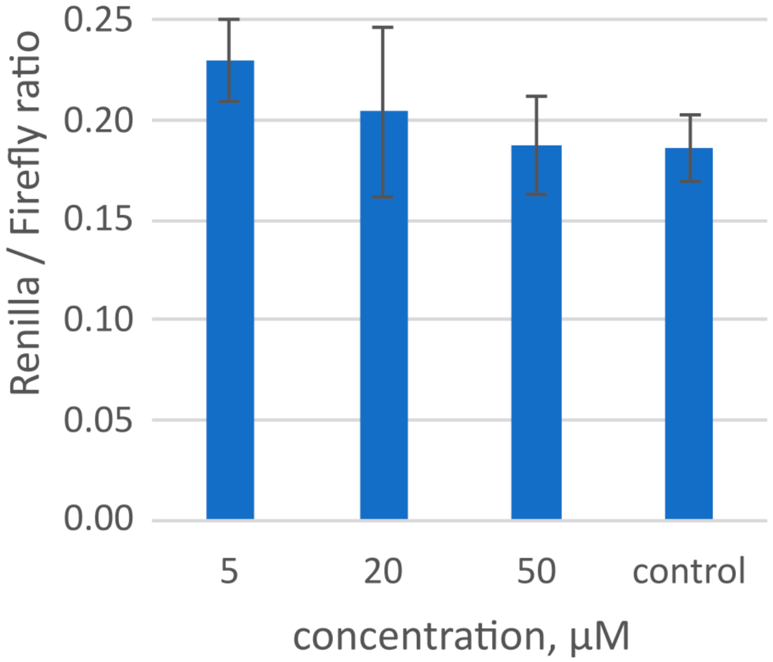

Figure S3. The dual luciferase reporter assay using the pC-Kit1 plasmid. HEK293 cells were transfected with the plasmid for 4 hours, followed by treatment with **5b** at a concentration of 5, 20 or 50 µM. No significant differences in the ability of **5b** to perturb relative gene-expressions compared with control, as assessed by the ratio of Renilla/Firefly expression levels, were observed. Error bars represent Standard error of Mean (SEM). Means were computed from N = 3 independent experiments.

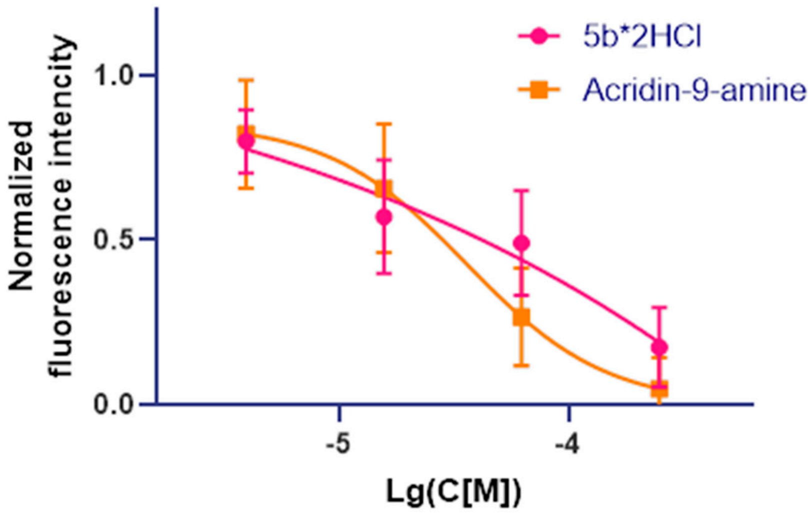

Figure S4. The EtBr displacement assay data for **5b** (4, 16, 62.5, 250 μM) demonstrates intercalation into DNA helix, Acridin-9-amine was used as a control sample . Means were computed from four independent experiments.

Table S4. Titration of DNA-targets with the benzo-imidazopyrimidinone derivatives.

Comp.	Comp. concentration, μM	ΔT_m °C±2 ($T_m^{lig} - T_m^{no\ lig}$)								
		22AG	cMyc	cKit1	Ct1	BclT	STAT	VEGF	22CTA	Hair
3a	1	0	0	1	-1	-1	-1	2	0	-1
	2	0	1	2	-1	-1	0	5	1	-1
	5	0	0	1	1	0	0	8	0	0
	10	1	0	2	2	0	0	2	1	-1
	20	1	1	3	2	0	1	1	1	-1
5a	1	1	1	6	4	1	2	-1	2	0
	2	2	1	7	4	1	5	-1	2	-1
	5	4	2	11	8	4	7	1	4	1
	10	6	3	14	9	6	10	13	5	2
	20	9	5	17	10	9	13	16	9	3
6a	1	0	1	4	2	0	1	4	2	-1
	2	1	1	5	3	0	2	6	1	-1
	5	3	1	8	5	2	6	10	2	0
	10	4	2	10	5	3	8	12	4	0
	20	6	2	12	6	5	13	15	5	1
7a	1	1	2	5	2	1	5	8	1	-1
	2	3	2	7	1	1	6	10	2	-1
	5	5	3	12	4	5	13	14	5	2
	10	8	6	15	5	6	14	18	7	3
	20	12	8	19	9	10	18	21	12	4

Table S5. Titration of DNA-targets with the naphtho-imidazopyrimidinone derivatives.

Comp.	Comp. concentration, µM	ΔT_m °C±2 ($T_m^{lig} - T_m^{no\ lig}$)								
		22AG	cMyc	cKit	Ct1	BclT	STAT	VEGF	22CTA	Hair
3b	1	1	1	2	-1	-1	1	1	0	-1
	2	2	1	4	0	0	2	2	1	-1
	5	4	2	8	-2	0	6	8	2	-3
	10	4	2	7	5	1	9	14	3	0
	20	6	4	11	6	4	10	18	5	0
5b	1	5	1	8	5	2	8	17	6	0
	2	7	1	11	6	4	11	20	8	1
	5	9	4	15	7	6	15	22	10	1
	10	13	7	21	16	14	24	27	15	7
	20	16	12	24	23	18	33	31	18	9
6b	1	4	1	10	9	2	12	16	3	1
	2	6	3	12	6	3	15	19	5	1
	5	7	3	15	6	2	17	22	7	0
	10	11	7	19	13	10	28	28	11	6
	20	23	16	35	30	24	23	>43	22	19
7b	1	4	1	10	9	2	12	16	3	1
	2	6	3	12	6	3	15	19	5	1
	5	7	3	15	6	2	17	22	7	0
	10	11	7	19	13	10	28	28	11	6
	20	14	>17	22	17	12	31	30	14	8

NMR spectra

Methyl 2-(7-hydroxy-1-oxobenzo[4,5]imidazo[1,2-c]pyrimidin-2(1H)-yl)acetate **2a**

^{1}H spectrum

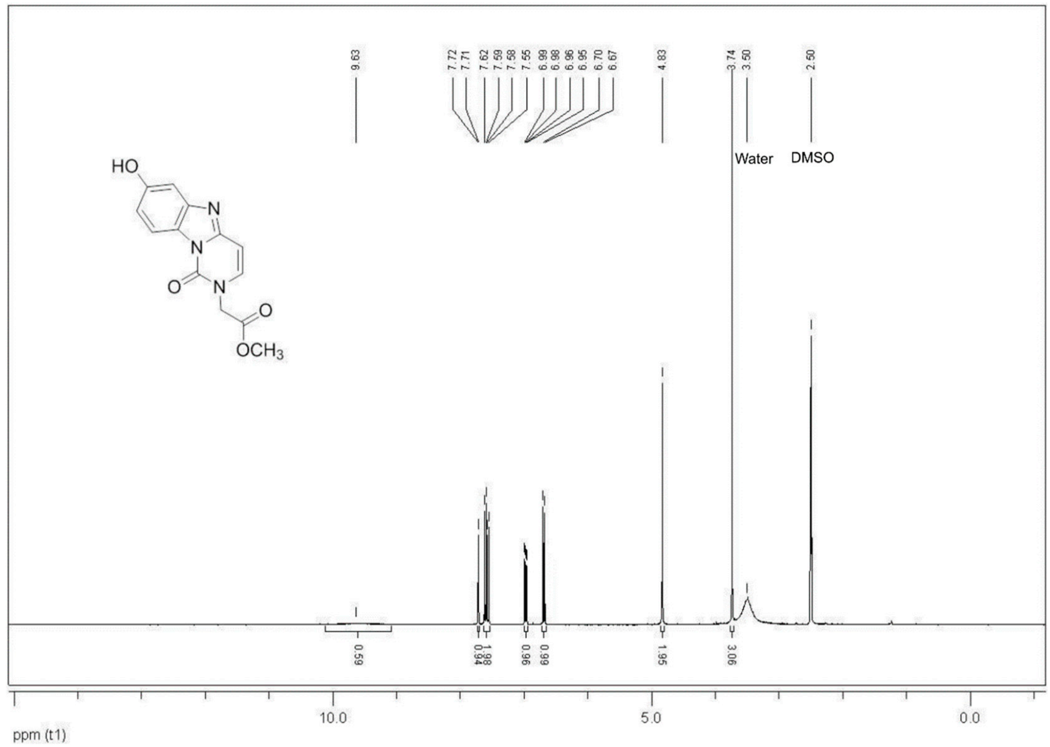

^{13}C spectrum

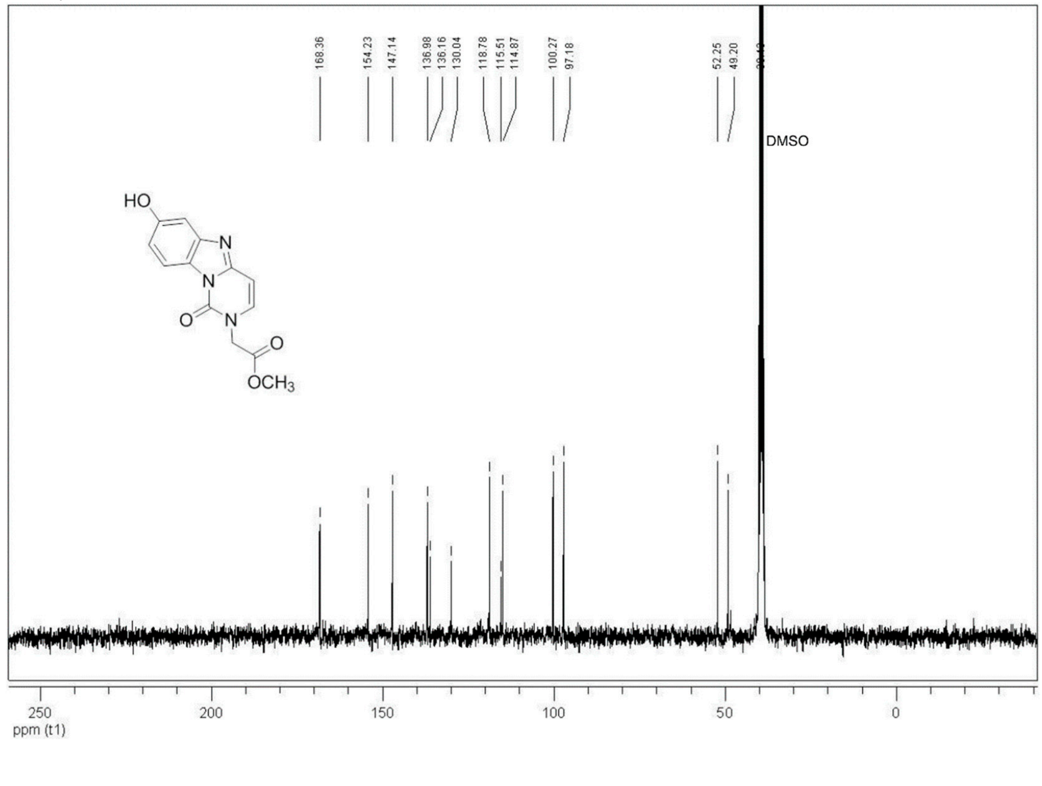

Methyl 2-(5-hydroxy-11-oxonaphtho[2',1':4,5]imidazo[1,2-c]pyrimidin-10(11H)-yl)acetate ***2b***

^{1}H spectrum

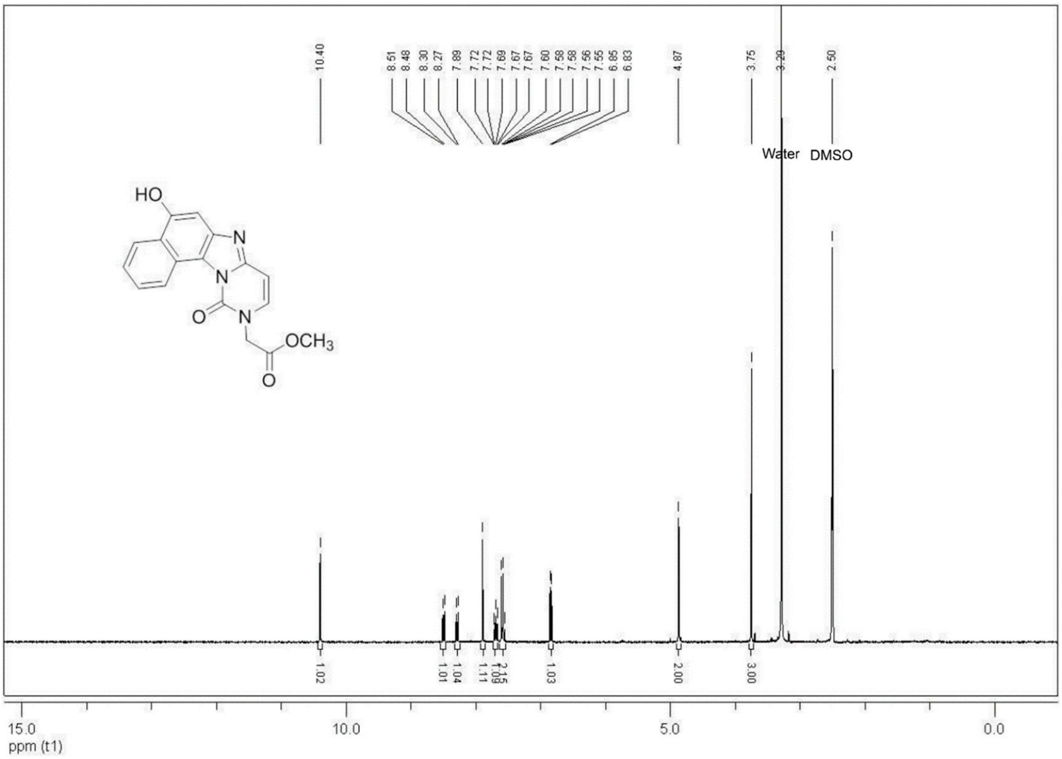

^{13}C spectrum

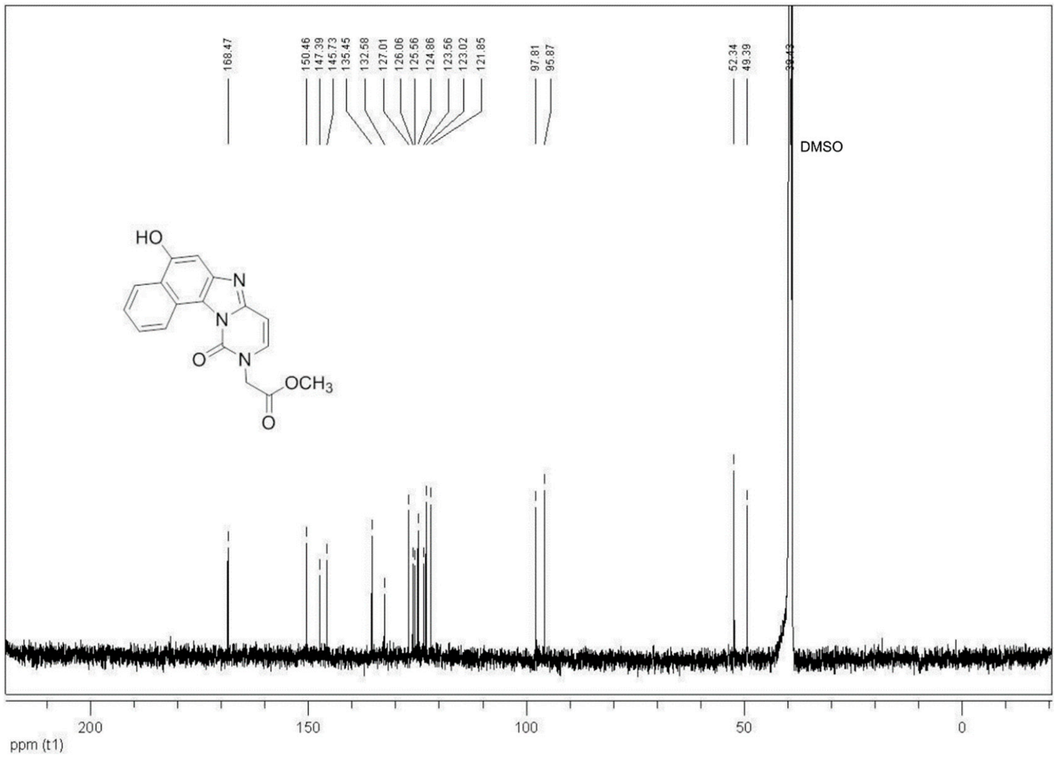

N-(2-Aminoethyl)-2-(7-hydroxy-1-oxobenzo[4,5]imidazo[1,2-c]pyrimidin-2(1H)-yl)acetamide **3a**

^{1}H spectrum

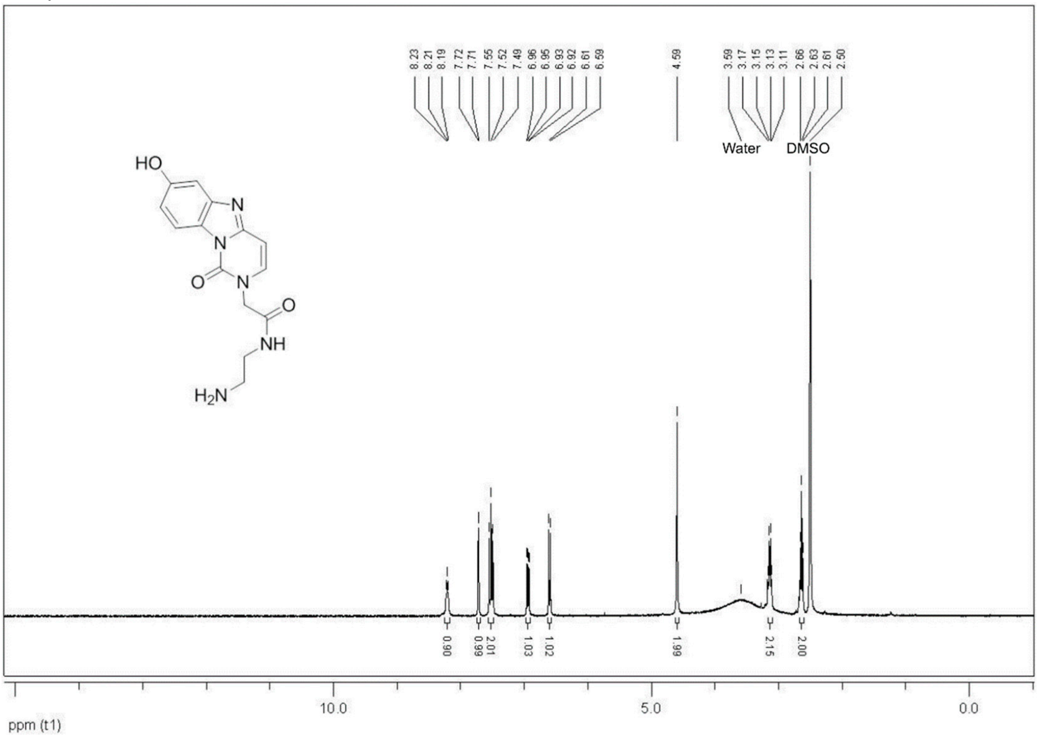

^{13}C spectrum

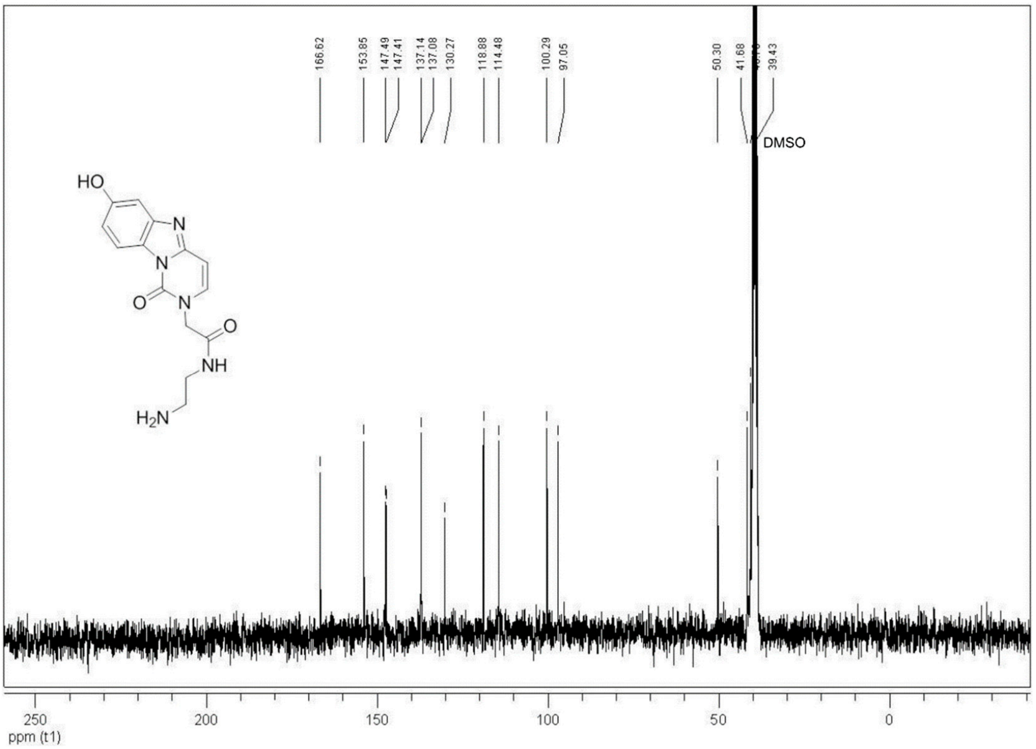

N-(2-Aminoethyl)-2-(5-hydroxy-11-oxonaphtho[2',1':4,5]imidazo[1,2-c]pyrimidin-10(11H)-yl)acetamide
3b
^{1}H spectrum

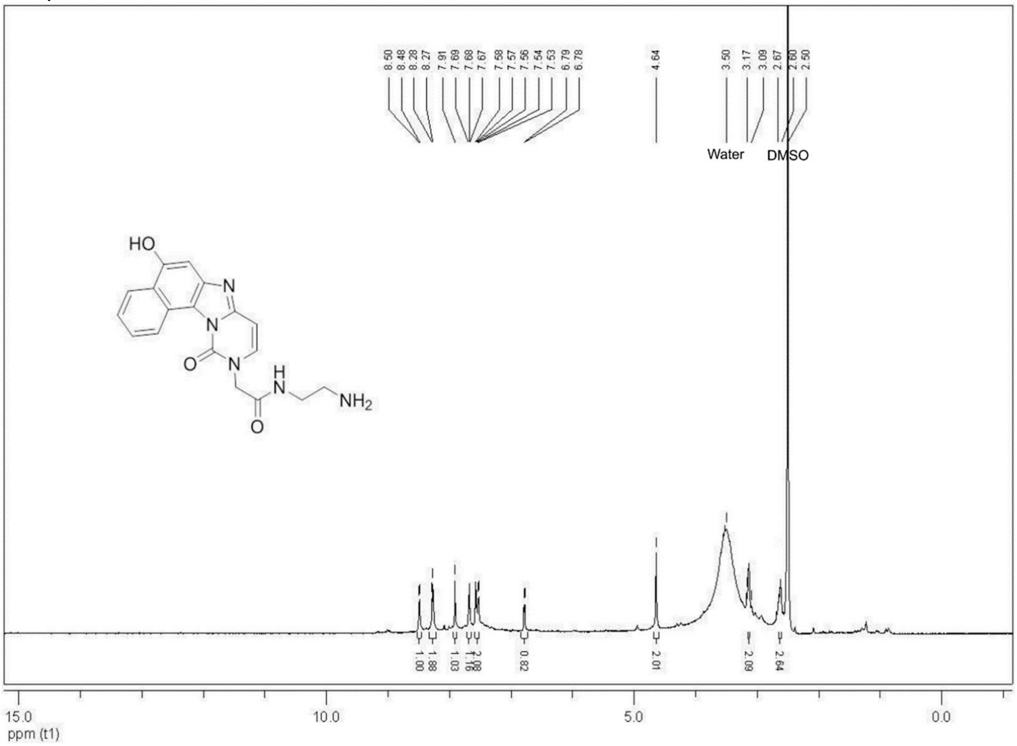

^{13}C spectrum

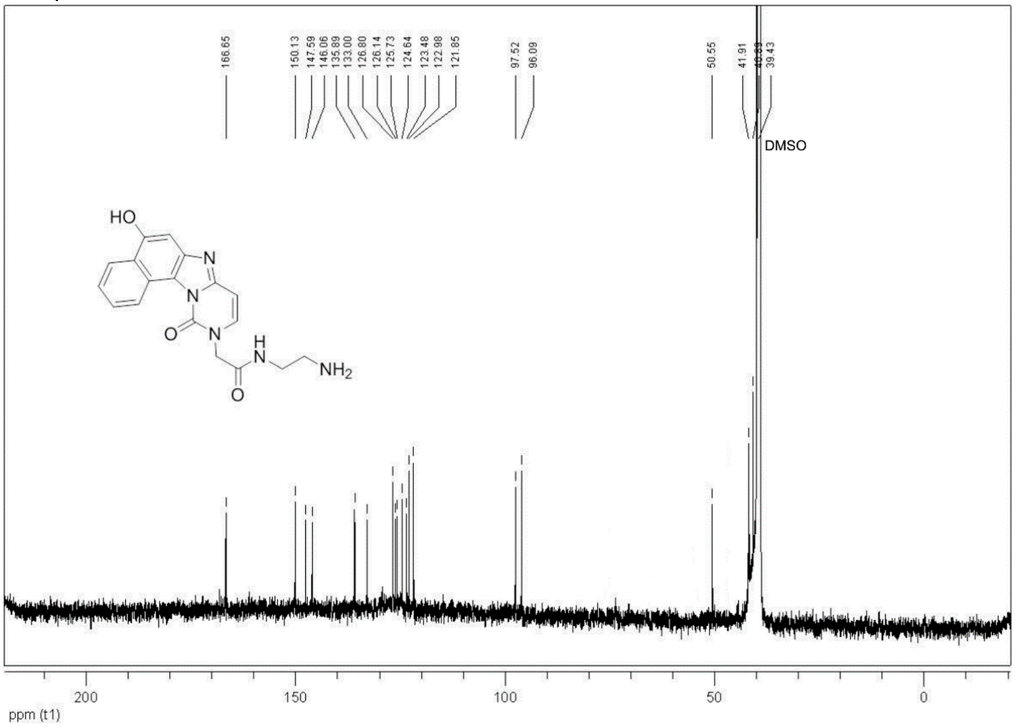

Methyl 2-(7-(2-methoxy-2-oxoethoxy)-1-oxobenzo[4,5]imidazo[1,2-c]pyrimidin-2(1H)-yl)acetate **4a**

^{1}H spectrum

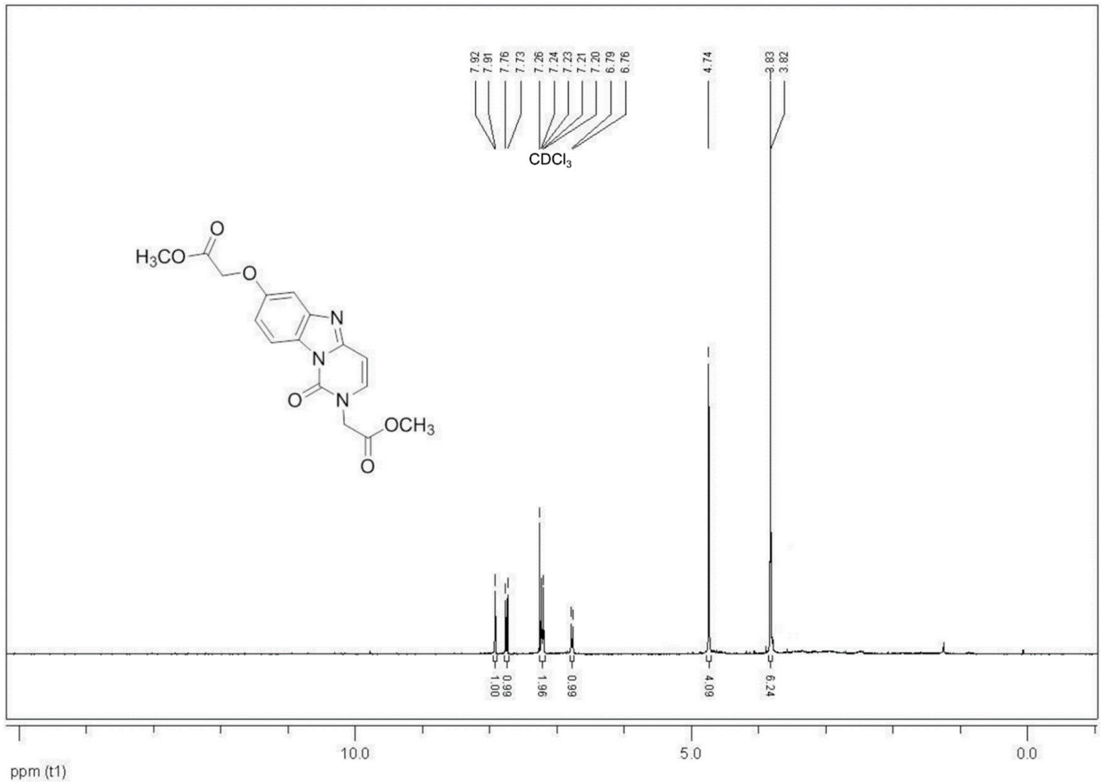

^{13}C spectrum

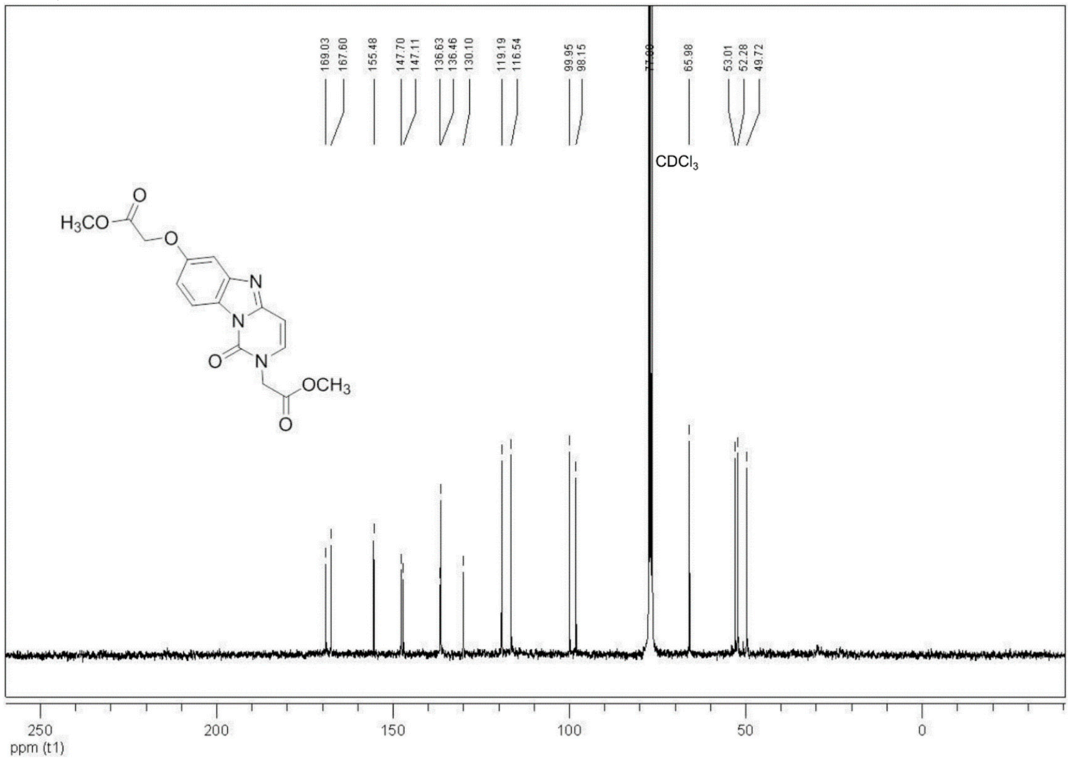

Methyl 2-(5-(2-methoxy-2-oxoethoxy)-11-oxonaphtho[2',1':4,5]imidazo[1,2-c]pyrimidin-10(11H)-yl)acetate **4b**

^{1}H spectrum

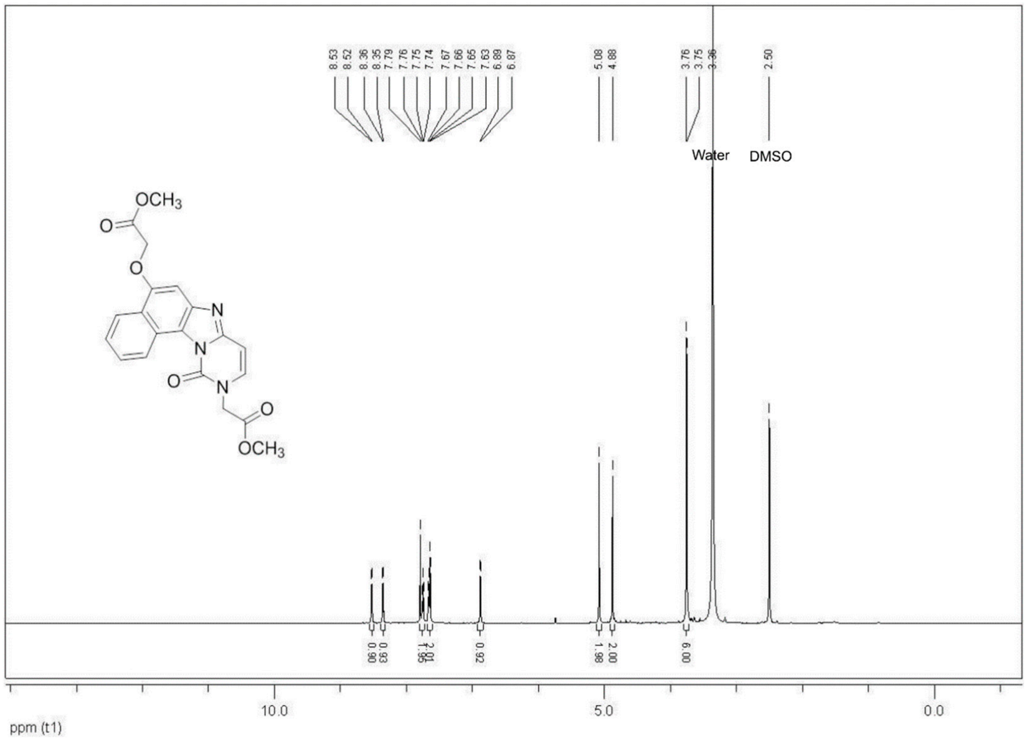

^{13}C spectrum

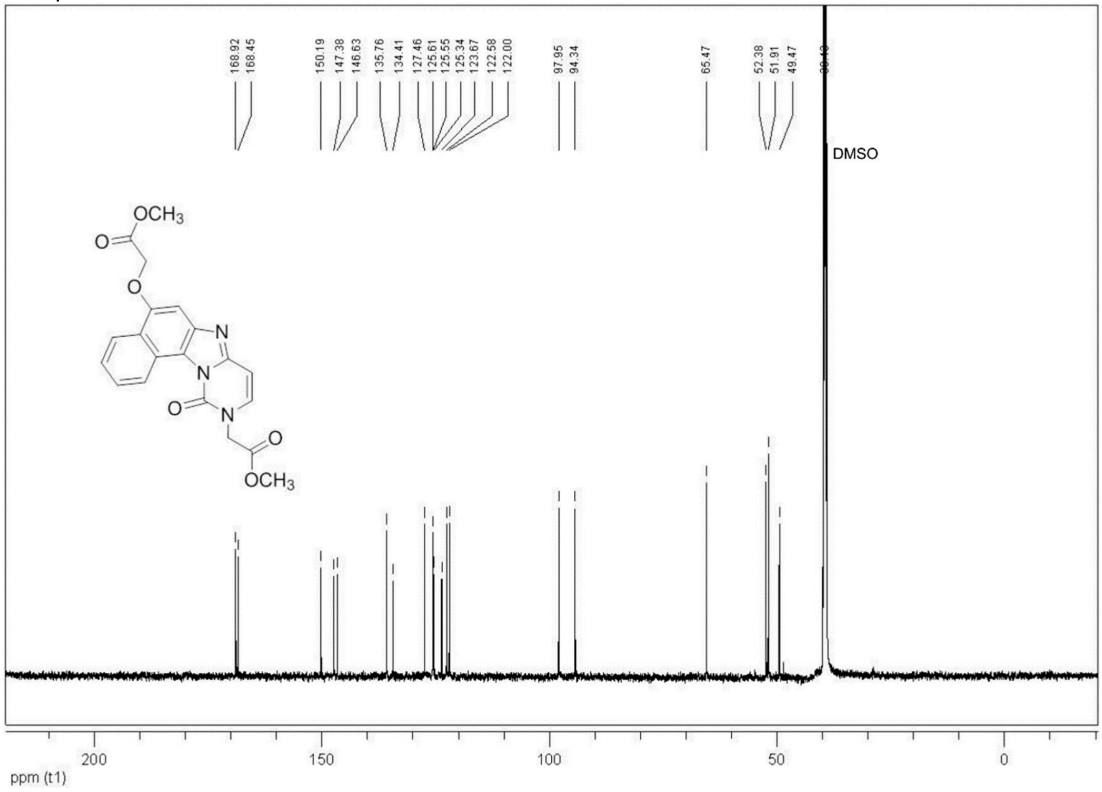

N-(2-Aminoethyl)-2-(7-(2-((2-aminoethyl)amino)-2-oxoethoxy)-1-oxobenzo[4,5]imidazo[1,2-c]pyrimidin-2(1H)-yl)acetamide **5a**

^{1}H spectrum

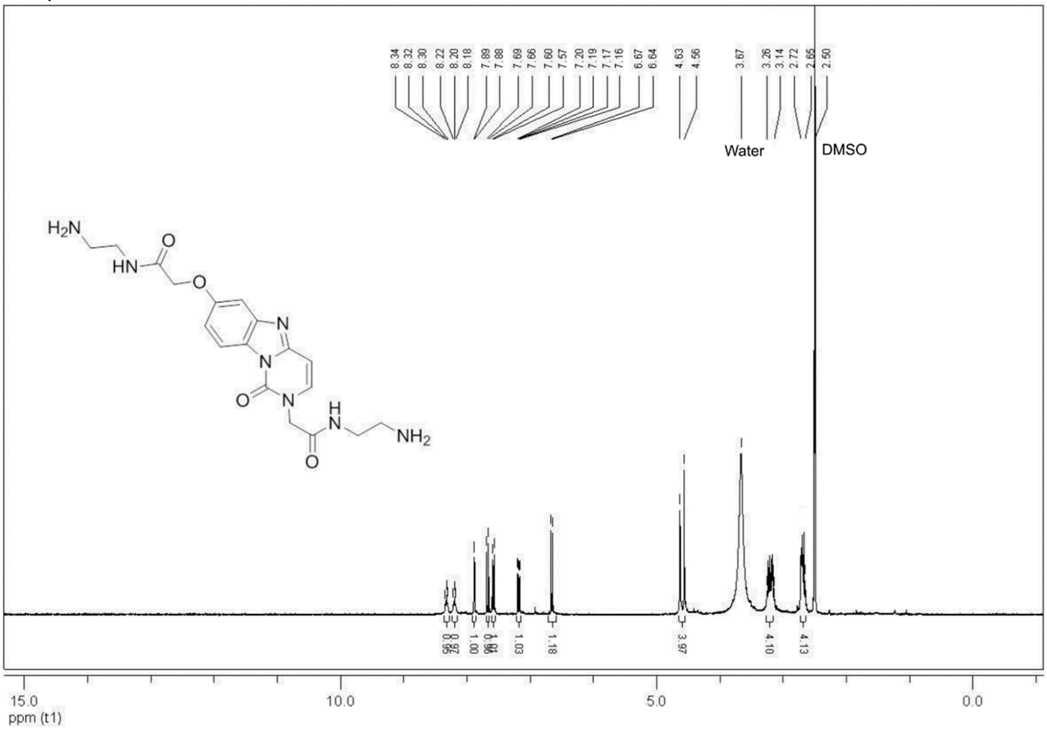

^{13}C spectrum

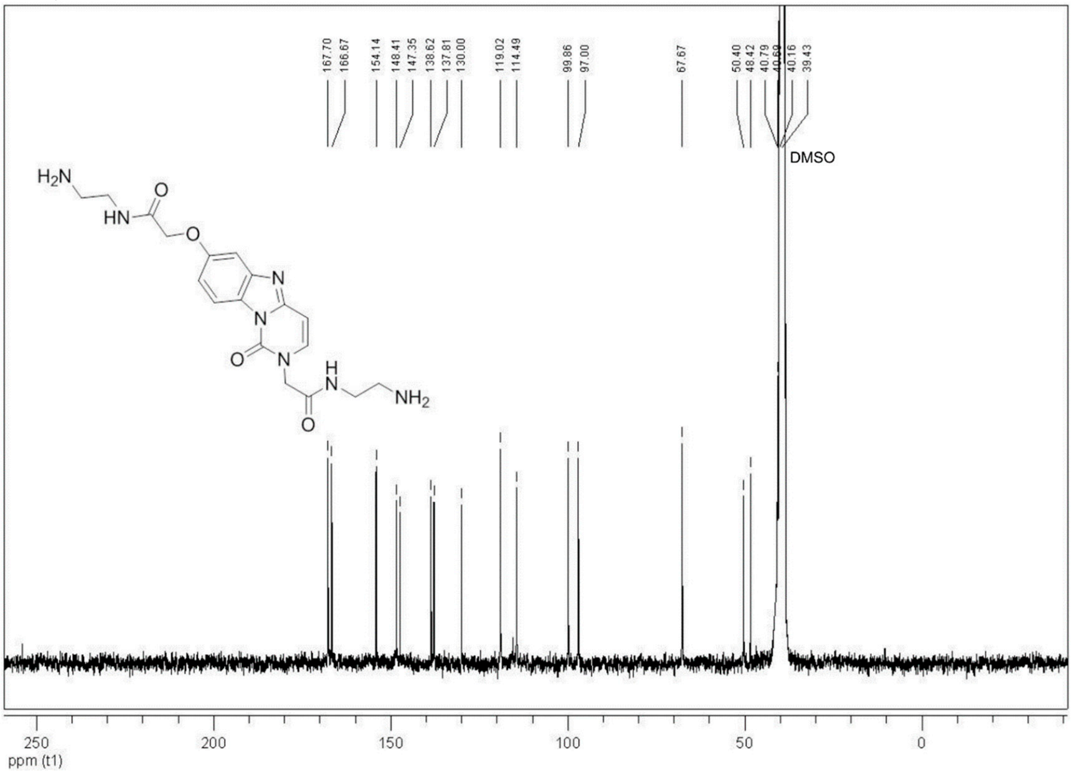

N-(2-Aminoethyl)-2-(5-(2-((2-aminoethyl)amino)-2-oxoethoxy)-11-oxonaphtho[2',1':4,5]imidazo[1,2-c]pyrimidin-10(11H)-yl)acetamide **5b**

^{1}H spectrum

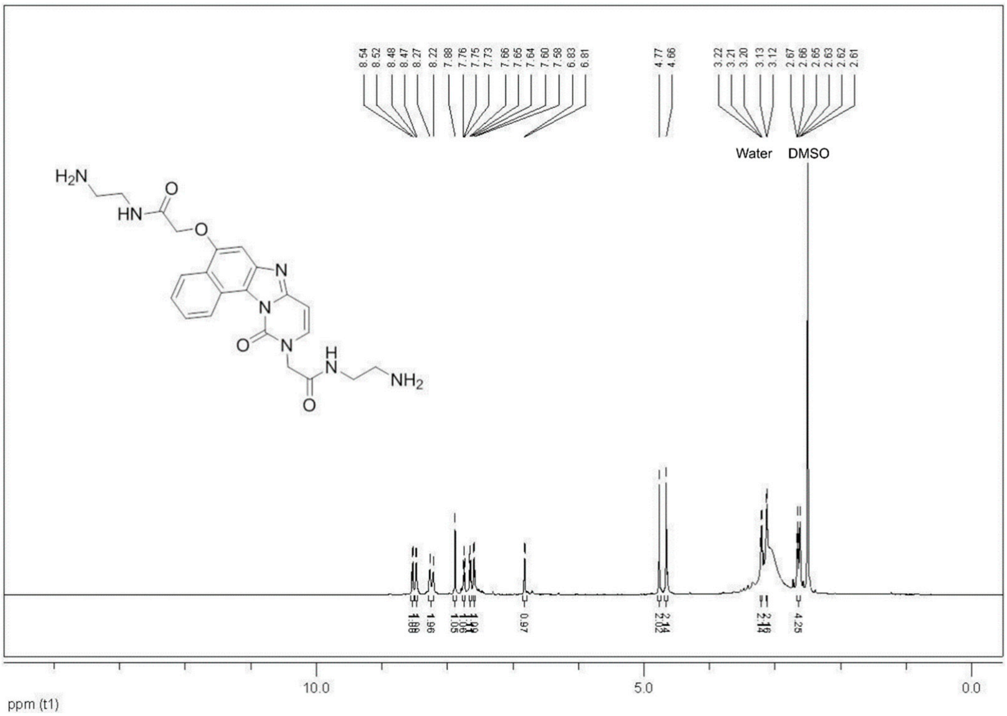

^{13}C spectrum

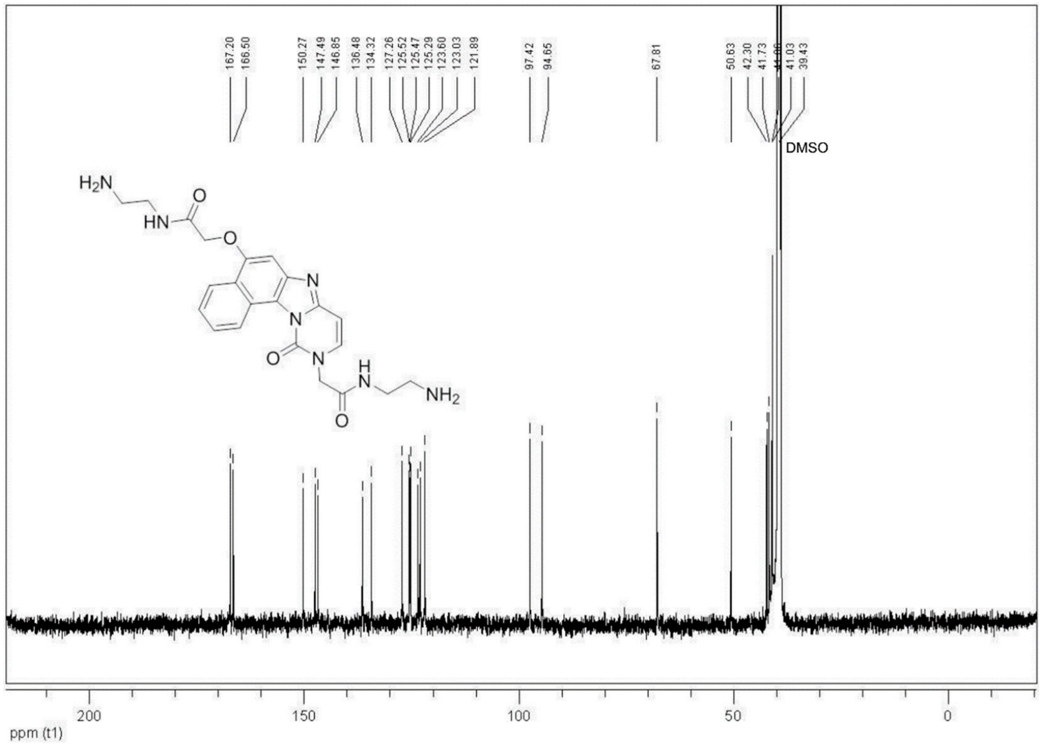

Ditrifluoroacetate salt of N-(2-(dimethylamino)ethyl)-2-(7-(2-((2-(dimethylamino)ethyl)amino)-2-oxoethoxy)-1-oxobenzo[4,5]imidazo[1,2-c]pyrimidin-2(1H)-yl)acetamide **6a**

^{1}H spectrum

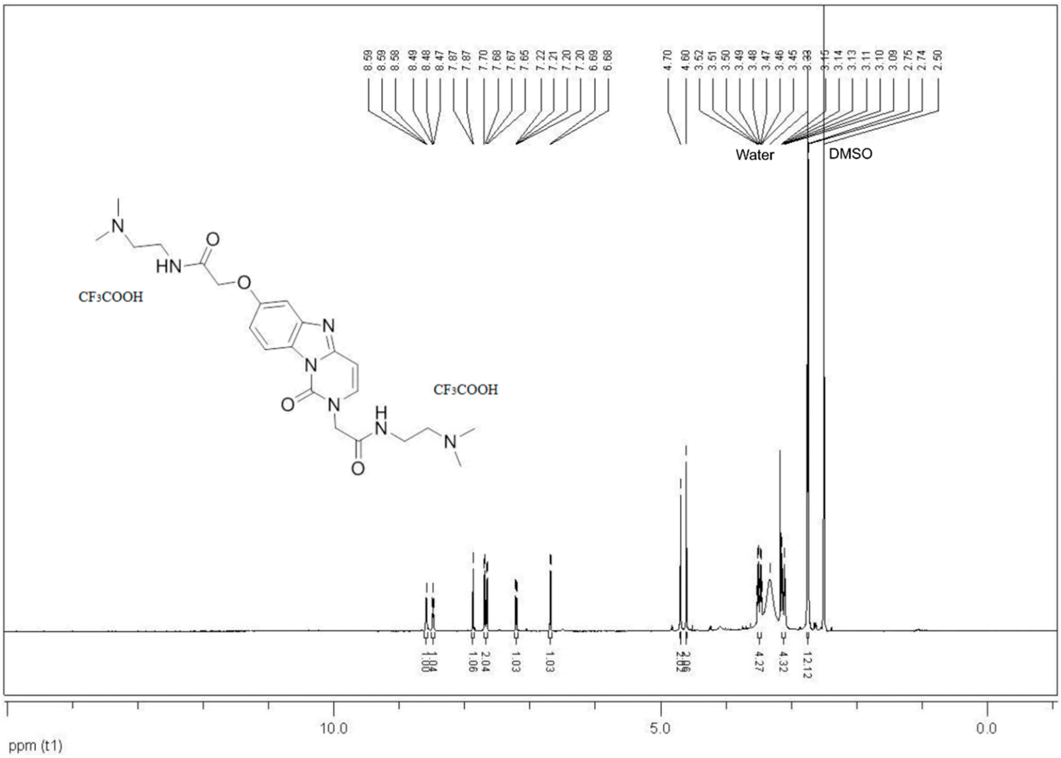

^{13}C spectrum

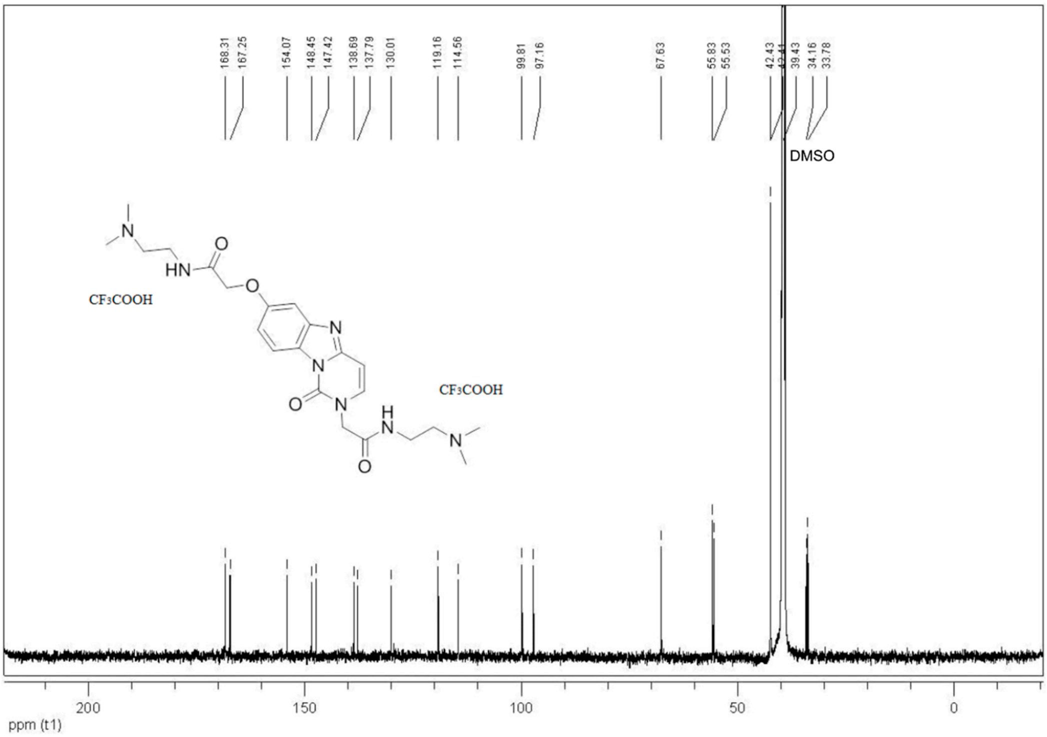

Ditrifluoroacetate salt of N-(2-(dimethylamino)ethyl)-2-(5-(2-((2-(dimethylamino)ethyl)amino)-2-oxoethoxy)-11-oxonaphtho[2',1':4,5]imidazo[1,2-c]pyrimidin-10(11H)-yl)acetamide **6b**

^{1}H spectrum

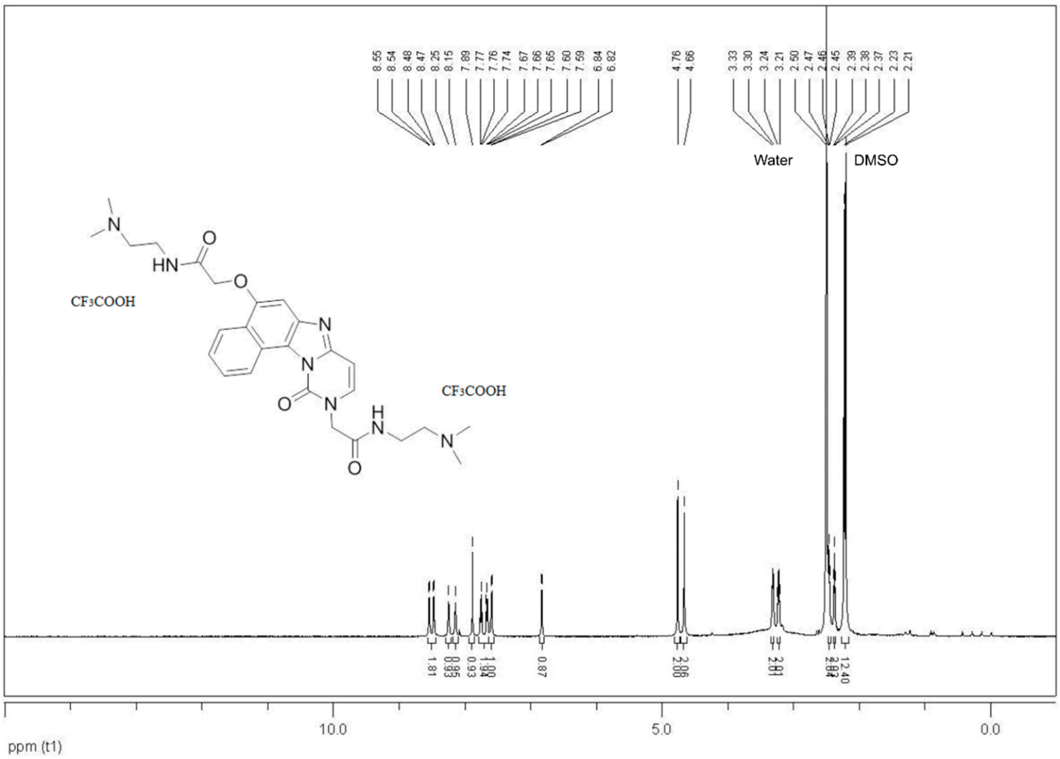

^{13}C spectrum

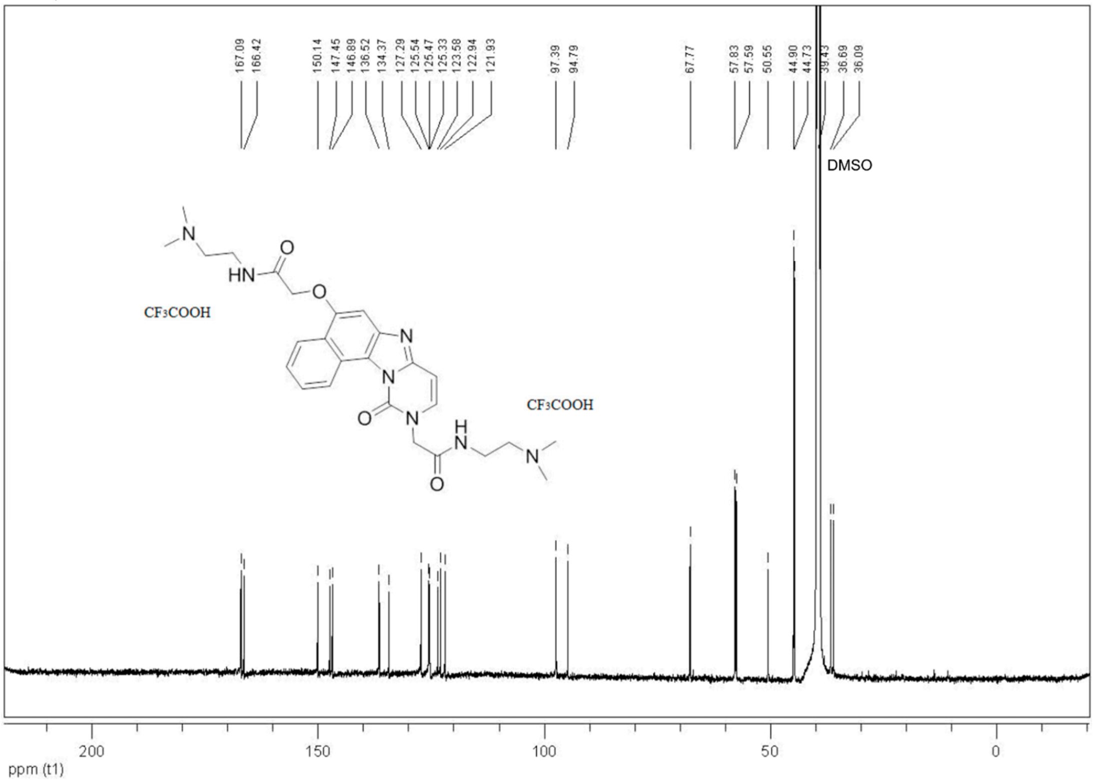

Ditrifluoroacetate salt of N-(2-guanidinoethyl)-2-(7-(2-((2-guanidinoethyl)amino)-2-oxoethoxy)-1-oxobenzo[4,5]imidazo[1,2-c]pyrimidin-2(1H)-yl)acetamide **7a**

^{1}H spectrum

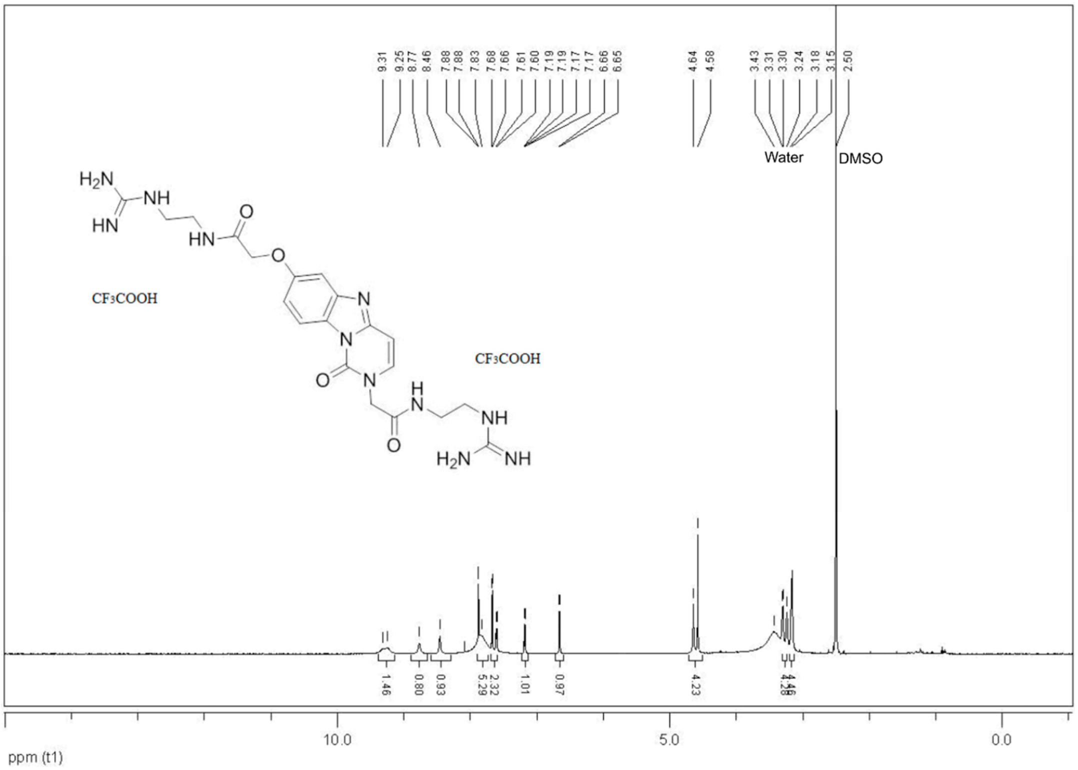

^{13}C spectrum

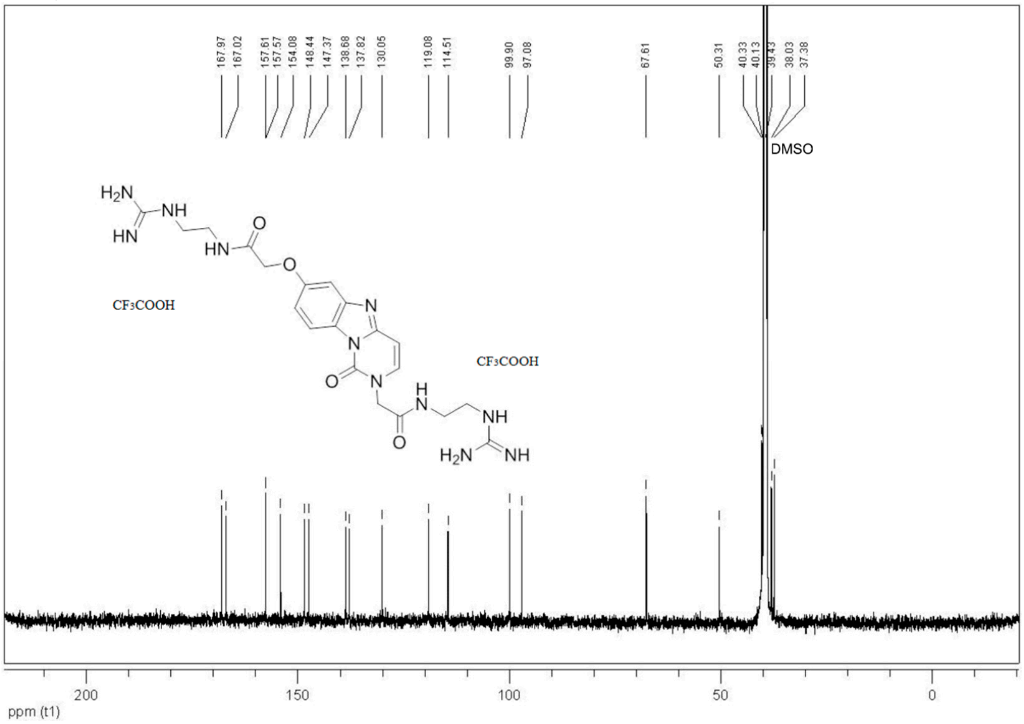

Ditrifluoroacetate salt of N-(2-guanidinoethyl)-2-(5-(2-((2-guanidinoethyl)amino)-2-oxoethoxy)-11-oxonaphtho[2',1':4,5]imidazo[1,2-c]pyrimidin-10(11H)-yl)acetamide **7b**

^{1}H spectrum

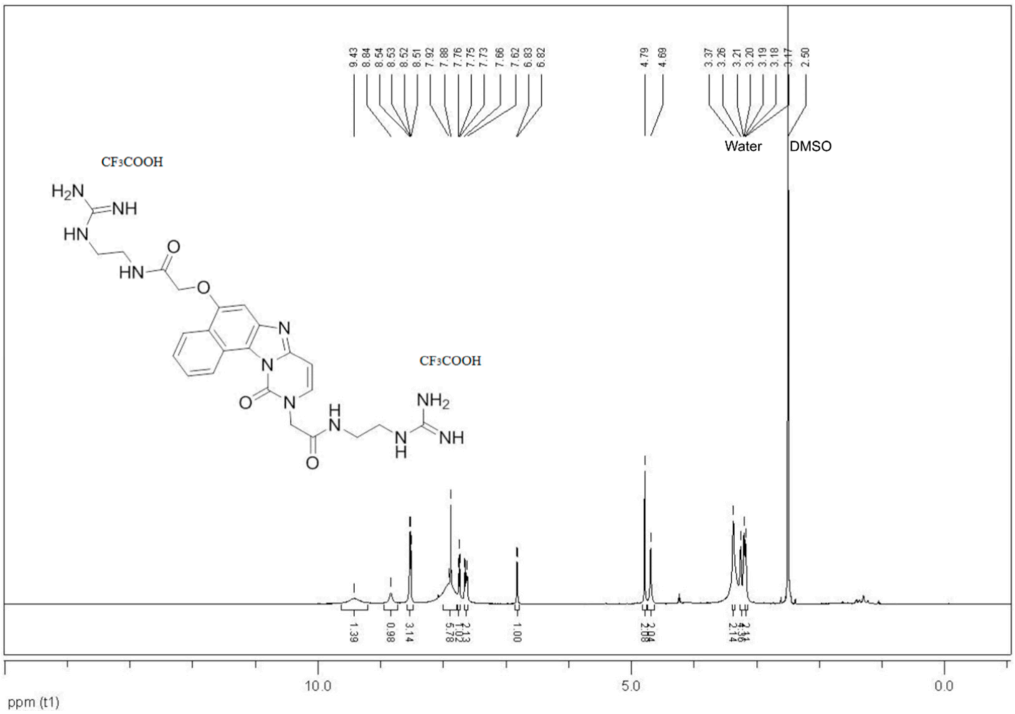

^{13}C spectrum

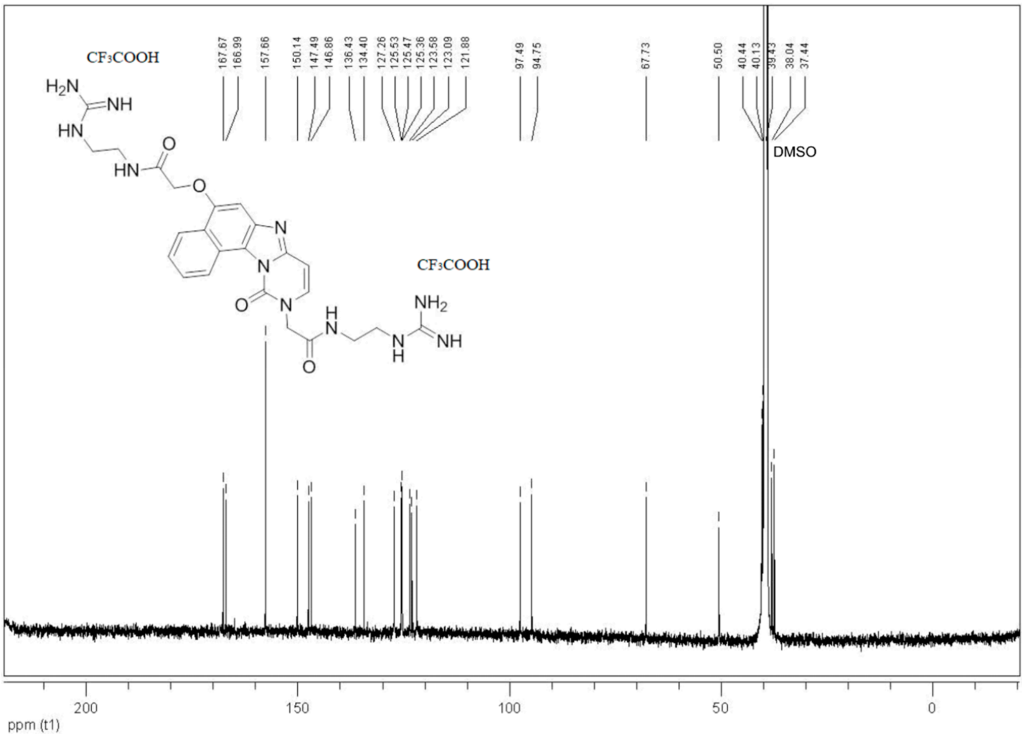

Dihydrochloride salt of N-(2-aminoethyl)-2-(5-(2-((2-aminoethyl)amino)-2-oxoethoxy)-11-oxonaphtho[2',1':4,5]imidazo[1,2-c]pyrimidin-10(11H)-yl)acetamide (**5b** × 2 HCl)

^{1}H spectrum

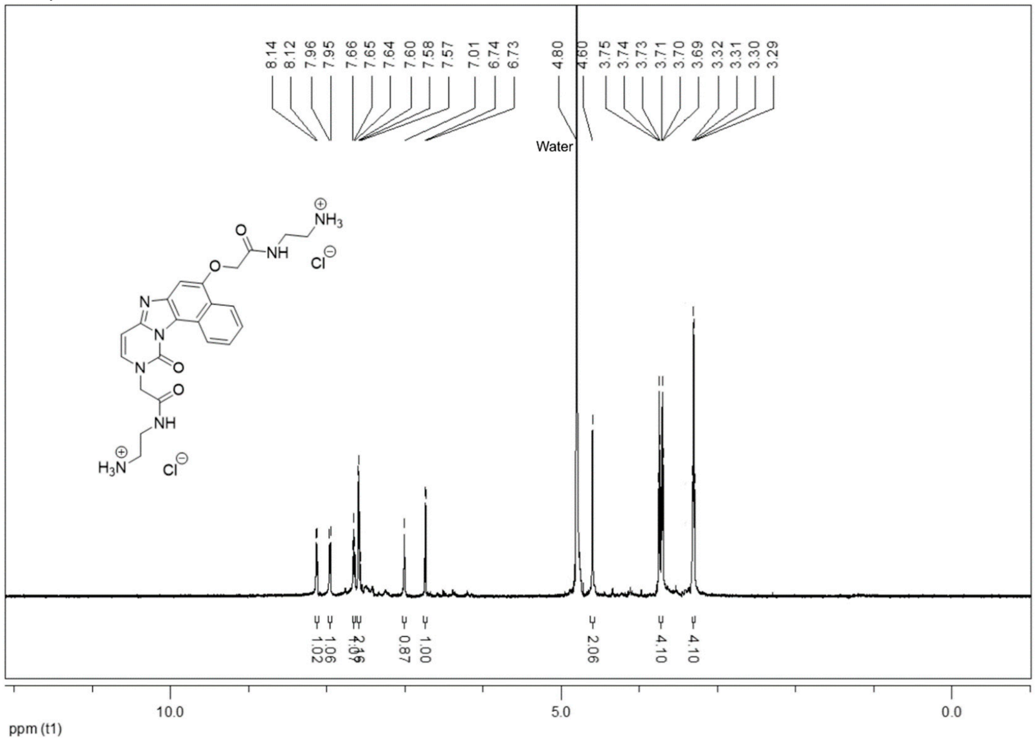

^{13}C spectrum

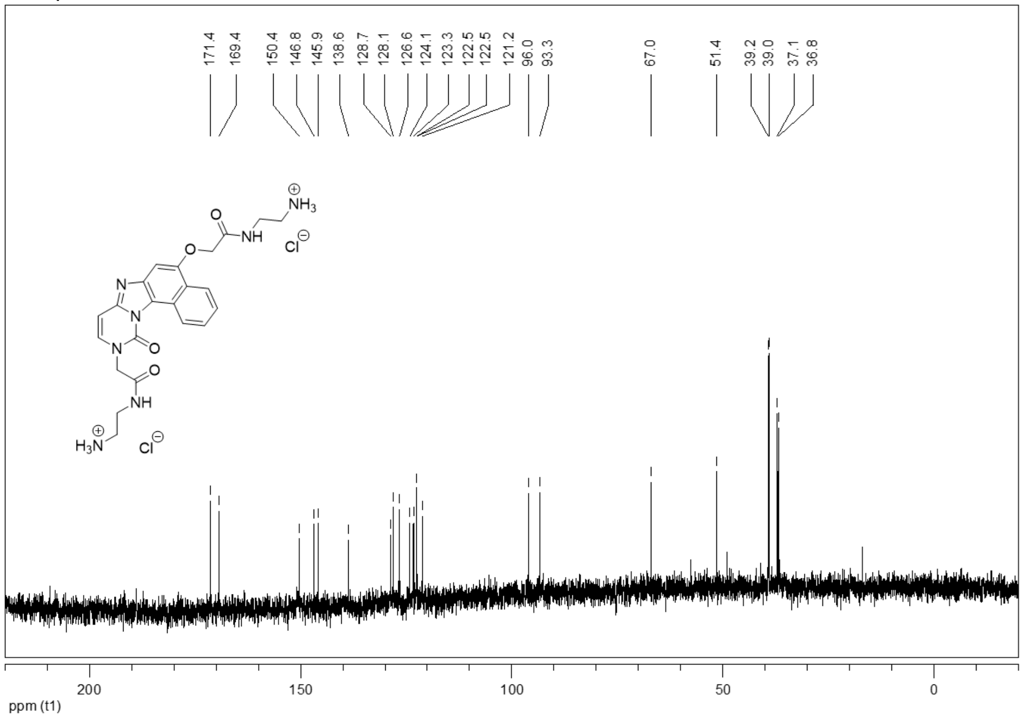

Analytical HPLC chromatograms

*N-(2-Aminoethyl)-2-(7-(2-((2-aminoethyl)amino)-2-oxoethoxy)-1-oxobenzo[4,5]imidazo[1,2-c]pyrimidin-2(1H)-yl)acetamide **5a***

```
=====================================================================
Acq. Operator   : SYSTEM
Sample Operator : SYSTEM
Acq. Instrument : Infinity II                   Location :   P1-C-02
Injection Date  : 28/10/2023 1:09:26 AM
                                              Inj Volume : 20.000 µl
Acq. Method     : C:\Chem32\1\Methods\New Objects.M
Last changed    : 28/10/2023 12:42:55 AM by SYSTEM
                  (modified after loading)
Analysis Method : C:\Chem32\1\Methods\Steps.M
Last changed    : 27/10/2023 8:41:47 PM by SYSTEM
                  (modified after loading)
Additional Info : Peak(s) manually integrated
```

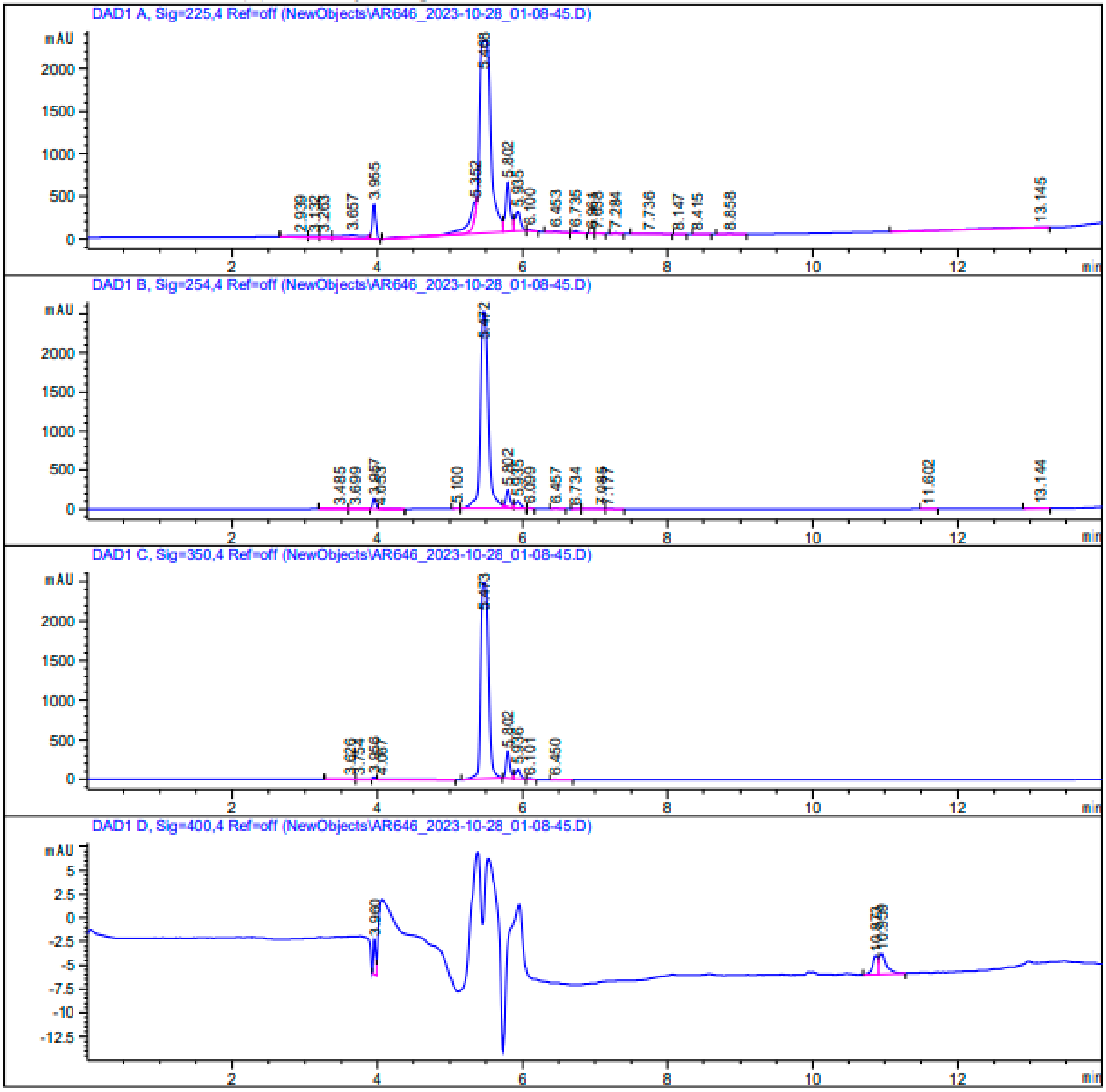

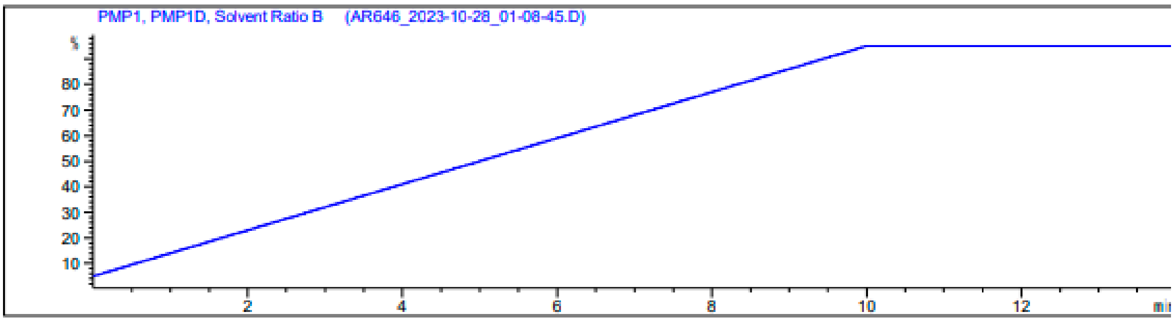

==

Fraction Information

==

No Fractions found.

==

==

===

Area Percent Report

===

Sorted By : Signal
Multiplier : 1.0000
Dilution : 1.0000
Use Multiplier & Dilution Factor with ISTDs

Signal 1: DAD1 A, Sig=225,4 Ref=off

Peak #	RetTime [min]	Type	Width [min]	Area [mAU*s]	Height [mAU]	Area %
1	2.939	BV E	0.2145	181.51338	11.11828	0.5268
2	3.132	VV E	0.1152	147.43823	17.70272	0.4279
3	3.263	VV E	0.1317	194.90529	19.99908	0.5657
4	3.657	VV E	0.2762	819.98602	37.33825	2.3800
5	3.955	VB R	0.0533	1429.65515	404.25992	4.1496
6	5.352	BV E	0.1861	2231.67773	148.78137	6.4775
7	5.468	VV R	0.1720	2.47424e4	2255.36597	71.8155
8	5.802	VV	0.0721	2789.94946	580.59247	8.0979
9	5.935	VB	0.0708	1108.39111	227.85310	3.2171
10	6.100	BB	0.0634	51.93304	11.77731	0.1507
11	6.453	VB R	0.0979	60.36980	8.59883	0.1752
12	6.735	BB	0.0675	90.57913	20.53106	0.2629
13	6.961	BV	0.0495	5.12052	1.59140	0.0149
14	7.038	VB	0.0673	20.98856	4.77364	0.0609
15	7.284	BB	0.0674	34.92330	8.25250	0.1014
16	7.736	BB	0.2542	76.70049	4.21681	0.2226
17	8.147	BB	0.0747	6.47171	1.33192	0.0188
18	8.415	BB	0.0686	11.02345	2.44707	0.0320
19	8.858	BB	0.1390	46.08928	4.84343	0.1338
20	13.145	BB	1.0592	402.65207	4.52899	1.1687

Totals : 3.44528e4 3775.90412

Signal 2: DAD1 B, Sig=254,4 Ref=off

Peak #	RetTime [min]	Type	Width [min]	Area [mAU*s]	Height [mAU]	Area %
1	3.485	BV E	0.1591	44.61381	3.68756	0.2152
2	3.699	VV E	0.1892	47.10183	3.54162	0.2272
3	3.957	VV R	0.0489	428.89532	135.66936	2.0687
4	4.053	VB E	0.1307	27.86984	2.93603	0.1344
5	5.100	BB	0.0627	13.80470	3.60004	0.0666
6	5.472	BV R	0.1143	1.86770e4	2524.18774	90.0837
7	5.802	VV E	0.0659	957.11896	223.71349	4.6164
8	5.935	VB E	0.0694	410.18930	86.43564	1.9784
9	6.099	BB	0.0491	13.62992	4.53060	0.0657
10	6.457	BB	0.0694	35.41249	7.73471	0.1708
11	6.734	BB	0.0591	8.23226	2.22743	0.0397
12	7.085	BV	0.1660	26.23187	2.04083	0.1265
13	7.177	VB	0.1048	16.72114	2.19737	0.0806
14	11.602	BB	0.0876	10.88652	1.93758	0.0525
15	13.144	BB	0.0911	15.24158	2.50148	0.0735

Totals : 2.07330e4 3006.94149

Signal 3: DAD1 C, Sig=350,4 Ref=off

Peak #	RetTime [min]	Type	Width [min]	Area [mAU*s]	Height [mAU]	Area %
1	3.626	BV E	0.1556	24.12868	2.10634	0.1195
2	3.754	VV E	0.1446	21.83938	2.01292	0.1081
3	3.956	VV R	0.0466	85.68097	28.86909	0.4242
4	4.067	VB E	0.4450	198.54945	5.74761	0.9830
5	5.473	BB	0.1157	1.78706e4	2491.61719	88.4769
6	5.802	BV	0.0641	1412.58142	342.84125	6.9937
7	5.936	VB	0.0655	533.39929	120.86829	2.6409
8	6.101	BB	0.0534	36.79314	10.90738	0.1822
9	6.450	BB	0.0787	14.45294	2.69012	0.0716

Totals : 2.01980e4 3007.66019

Signal 4: DAD1 D, Sig=400,4 Ref=off

Peak #	RetTime [min]	Type	Width [min]	Area [mAU*s]	Height [mAU]	Area %
1	3.960	BV	0.0362	8.24387	3.66410	24.5355
2	10.873	BV	0.0759	10.30634	2.00623	30.6738
3	10.959	VB	0.0991	15.04958	2.16576	44.7907

Totals : 33.59980 7.83608

N-(2-Aminoethyl)-2-(5-(2-((2-aminoethyl)amino)-2-oxoethoxy)-11-oxonaphtho[2',1':4,5]imidazo[1,2-c]pyrimidin-10(11H)-yl)acetamide **5b**

===

Acq. Operator : SYSTEM

Sample Operator : SYSTEM

Acq. Instrument : Infinity II Location : P1-F-01

Injection Date : 28/10/2023 2:52:10 AM

Inj Volume : 20.000 µl

Acq. Method : C:\Chem32\1\Methods\New Objects.M

Last changed : 28/10/2023 2:46:22 AM by SYSTEM

(modified after loading)

Analysis Method : C:\Chem32\1\Methods\Steps.M

Last changed : 13/10/2023 5:42:38 PM by SYSTEM

Additional Info : Peak(s) manually integrated

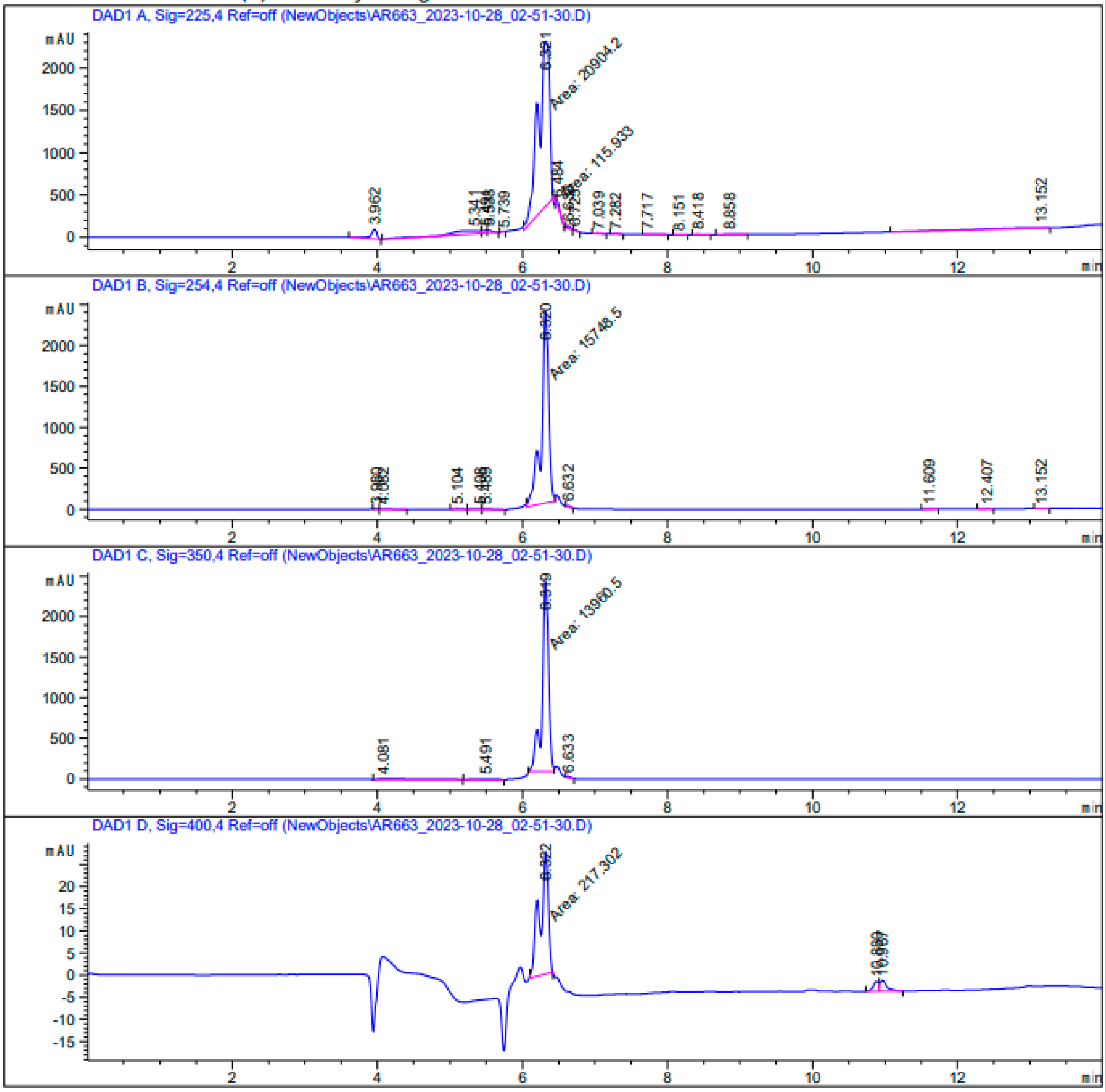

Signal 1: DAD1 A, Sig=225,4 Ref=off

Peak #	RetTime [min]	Type	Width [min]	Area [mAU*s]	Height [mAU]	Area %
1	3.962	BB	0.0905	708.56458	114.04625	2.9529
2	5.341	BV	0.4444	1263.27686	35.06133	5.2646
3	5.491	VV	0.0609	133.79697	30.66529	0.5576
4	5.538	VB	0.0685	141.94795	29.31156	0.5916
5	5.739	BB	0.0449	12.44818	4.70473	0.0519
6	6.321	MM	0.1792	2.09042e4	1944.30432	87.1156
7	6.484	MM	0.0377	115.93272	51.31052	0.4831
8	6.633	BB	0.0486	80.95962	27.31433	0.3374
9	6.725	BB	0.0523	22.83165	6.94644	0.0951
10	7.039	BB	0.0716	24.81981	5.40698	0.1034
11	7.282	BB	0.0673	38.91401	9.21063	0.1622
12	7.717	BB	0.2265	22.77489	1.32534	0.0949
13	8.151	BB	0.0754	7.39559	1.45187	0.0308
14	8.418	BB	0.0686	13.40351	2.86567	0.0559
15	8.858	BB	0.1407	49.29311	5.10088	0.2054
16	13.152	BB	1.1234	455.36929	4.81485	1.8977
Totals :				2.39959e4	2273.84098	

Signal 2: DAD1 B, Sig=254,4 Ref=off

Peak #	RetTime [min]	Type	Width [min]	Area [mAU*s]	Height [mAU]	Area %
1	3.980	BV	0.0411	24.50242	9.83030	0.1519
2	4.082	VB	0.1476	22.71611	2.17994	0.1408
3	5.104	BV	0.0993	52.19427	7.49082	0.3236
4	5.408	VV	0.1207	63.09685	6.89979	0.3912
5	5.489	VB	0.1820	138.79689	9.64108	0.8605
6	6.320	MM	0.1121	1.57485e4	2341.53101	97.6334
7	6.632	BB	0.0504	37.90651	12.13863	0.2350
8	11.609	BB	0.0844	13.30799	2.41085	0.0825
9	12.407	BV	0.1024	16.02007	2.38374	0.0993
10	13.152	BB	0.0832	13.19912	2.43758	0.0818
Totals :				1.61302e4	2396.94374	

Signal 3: DAD1 C, Sig=350,4 Ref=off

Peak #	RetTime [min]	Type	Width [min]	Area [mAU*s]	Height [mAU]	Area %
1	4.081	BB	0.5096	555.07172	14.09029	3.7660
2	5.491	BB	0.2223	180.36392	10.10016	1.2237
3	6.319	MM	0.0996	1.39605e4	2336.38550	94.7178
4	6.633	BB	0.0530	43.10951	12.90167	0.2925
Totals :				1.47391e4	2373.47763	

Signal 4: DAD1 D, Sig=400,4 Ref=off

Peak #	RetTime [min]	Type	Width [min]	Area [mAU*s]	Height [mAU]	Area %
1	6.322	MM	0.1314	217.30229	27.55509	88.8431
2	10.880	BV	0.0750	11.43287	2.26026	4.6743
3	10.967	VB	0.0954	15.85590	2.39086	6.4826
Totals :				244.59105	32.20621	

Ditrifluoroacetate salt of N-(2-(dimethylamino)ethyl)-2-(7-(2-((2-(dimethylamino)ethyl)amino)-2-oxoethoxy)-1-oxobenzo[4,5]imidazo[1,2-c]pyrimidin-2(1H)-yl)acetamide **6a**

```
=====================================================================
Acq. Operator   : SYSTEM
Sample Operator : SYSTEM
Acq. Instrument : Infinity II                   Location :   P1-B-03
Injection Date  : 14/7/2023 8:40:42 PM
                                              Inj Volume : 100.000 µl
Acq. Method     : C:\Chem32\1\Methods\New Objects.M
Last changed    : 14/7/2023 7:56:22 PM by SYSTEM
                  (modified after loading)
Analysis Method : C:\Chem32\1\Methods\Steps.M
Last changed    : 14/7/2023 9:47:06 PM by SYSTEM
                  (modified after loading)
```

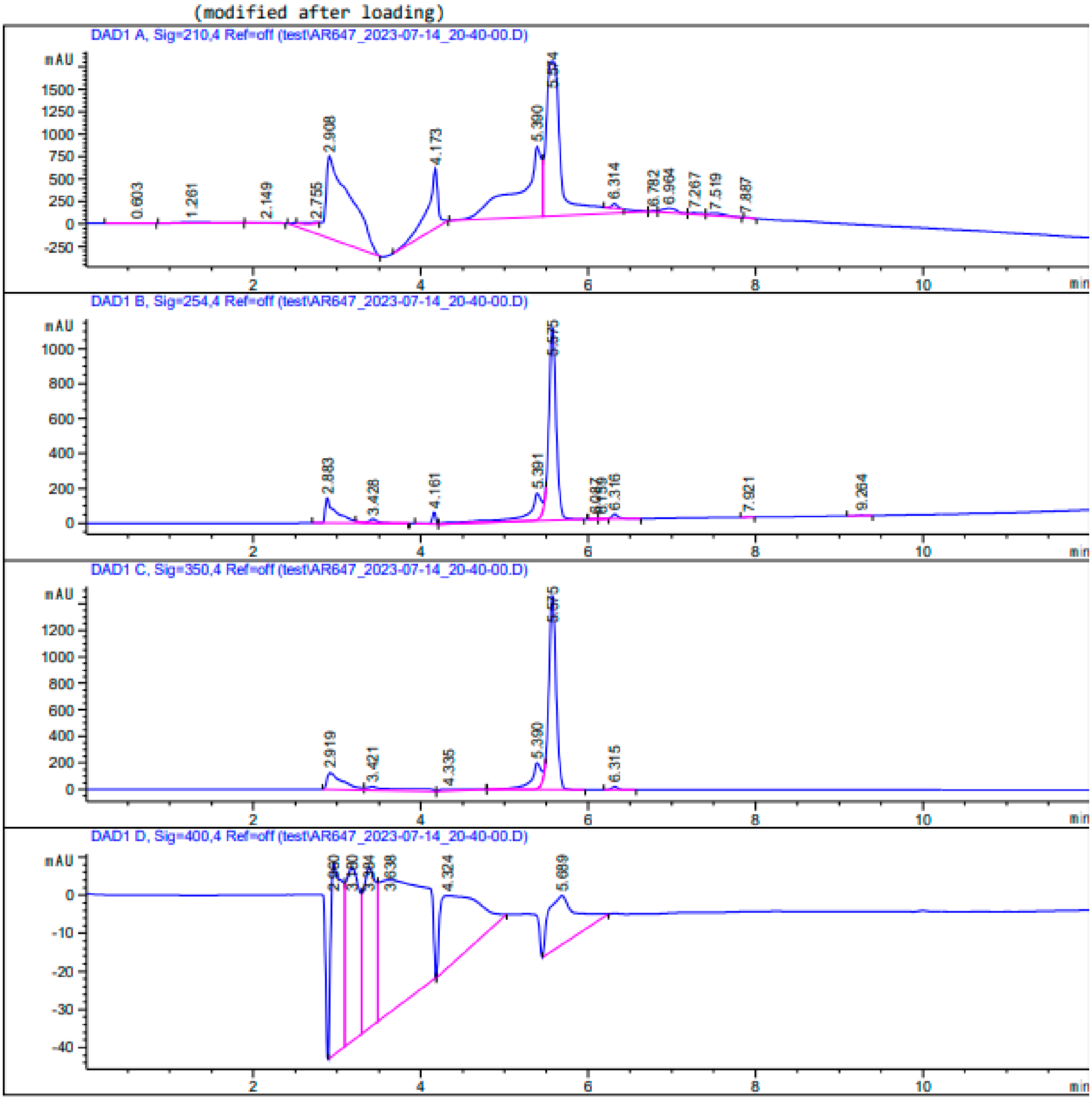

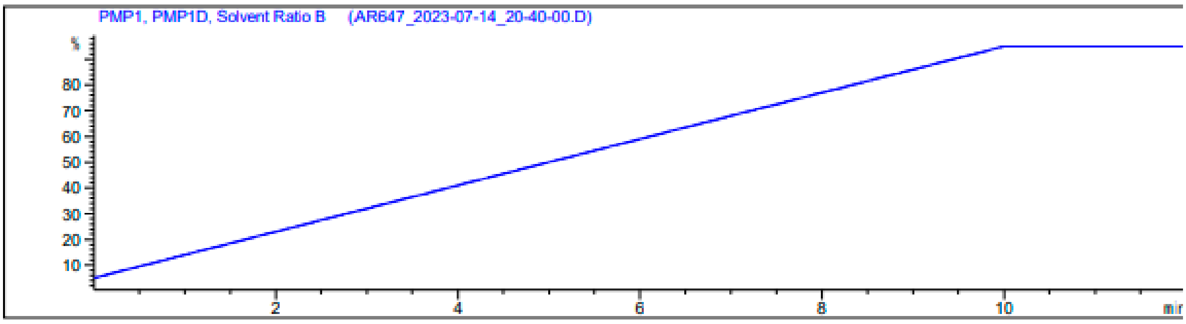

===

Fraction Information

===

No Fractions found.

===

===

===

Area Percent Report

===

Sorted By : Signal
Multiplier : 1.0000
Dilution : 1.0000
Use Multiplier & Dilution Factor with ISTDs

Signal 1: DAD1 A, Sig=210,4 Ref=off

Peak #	RetTime [min]	Type	Width [min]	Area [mAU*s]	Height [mAU]	Area %
1	0.603	BB	0.2766	39.97421	2.00521	0.0617
2	1.261	BB	0.4099	304.24753	9.80759	0.4700
3	2.149	BB	0.1966	19.43455	1.25569	0.0300
4	2.755	BV X	0.2737	168.32822	7.62129	0.2600
5	2.908	VB R	0.2778	2.03001e4	911.50598	31.3582
6	4.173	BB	0.1183	5889.60938	658.78400	9.0979
7	5.390	BV	0.2353	1.47732e4	778.94836	22.8206
8	5.574	VV R	0.1897	2.17518e4	1718.72400	33.6007
9	6.314	VB E	0.0723	251.17648	52.02405	0.3880
10	6.782	BV	0.0648	52.70024	12.59033	0.0814
11	6.964	VV	0.1810	605.55212	49.41012	0.9354
12	7.267	VV	0.1753	183.40521	14.72562	0.2833
13	7.519	VB	0.2066	378.20114	26.79955	0.5842
14	7.887	BBA	0.0968	18.50898	2.88589	0.0286

Totals : 6.47362e4 4247.08769

Signal 2: DAD1 B, Sig=254,4 Ref=off

Peak #	RetTime [min]	Type	Width [min]	Area [mAU*s]	Height [mAU]	Area %
1	2.883	BV R	0.1204	1338.68945	144.10365	12.6057
2	3.428	VB E	0.1051	163.99196	22.49311	1.5442
3	4.161	BB	0.0468	200.67796	67.32065	1.8897
4	5.391	BV E	0.1572	1855.43066	151.19574	17.4715
5	5.575	VB R	0.0949	6871.58203	1099.58862	64.7058
6	6.087	BV E	0.0641	10.22171	2.47849	0.0963
7	6.159	VV E	0.0683	10.95023	2.35219	0.1031
8	6.316	VV R	0.0794	148.59584	27.33676	1.3992
9	7.921	BB	0.0828	5.22790	1.03666	0.0492
10	9.264	BB	0.0964	14.35843	2.19262	0.1352
Totals :				1.06197e4	1520.09848	

Signal 3: DAD1 C, Sig=350,4 Ref=off

Peak #	RetTime [min]	Type	Width [min]	Area [mAU*s]	Height [mAU]	Area %
1	2.919	BV	0.1776	1639.05273	124.50528	11.9400
2	3.421	VB	0.3808	900.56488	29.11974	6.5603
3	4.335	BB	0.2981	318.17529	14.60966	2.3178
4	5.390	BV E	0.1124	1650.75635	195.68581	12.0252
5	5.575	VB R	0.0945	9089.43066	1463.60291	66.2135
6	6.315	BB	0.0736	129.47760	26.24174	0.9432
Totals :				1.37275e4	1853.76513	

Signal 4: DAD1 D, Sig=400,4 Ref=off

Peak #	RetTime [min]	Type	Width [min]	Area [mAU*s]	Height [mAU]	Area %
1	2.960	BV	0.1290	478.30270	50.27729	13.5782
2	3.180	VV	0.1620	521.64355	45.41421	14.8085
3	3.384	VV	0.1470	452.63565	42.23880	12.8495
4	3.638	VB	0.4634	1230.80078	34.73159	34.9403
5	4.324	BB	0.3736	536.42633	18.76206	15.2282
6	5.689	BB	0.3109	302.77487	12.75481	8.5952
Totals :				3522.58389	204.17876	

Ditrifluoroacetate salt of N-(2-(dimethylamino)ethyl)-2-(5-(2-((2-(dimethylamino)ethyl)amino)-2-oxoethoxy)-11-oxonaphtho[2',1':4,5]imidazo[1,2-c]pyrimidin-10(11H)-yl)acetamide **6b**

```
=====================================================================
Acq. Operator   : SYSTEM
Sample Operator : SYSTEM
Acq. Instrument : Infinity II                 Location :   P1-E-01
Injection Date  : 28/10/2023 1:54:58 AM
                                            Inj Volume : 100.000 µl
Acq. Method     : C:\Chem32\1\Methods\New Objects.M
Last changed    : 28/10/2023 1:28:42 AM by SYSTEM
                  (modified after loading)
Analysis Method : C:\Chem32\1\Methods\Steps.M
Last changed    : 27/10/2023 8:41:47 PM by SYSTEM
                  (modified after loading)
Additional Info : Peak(s) manually integrated
```

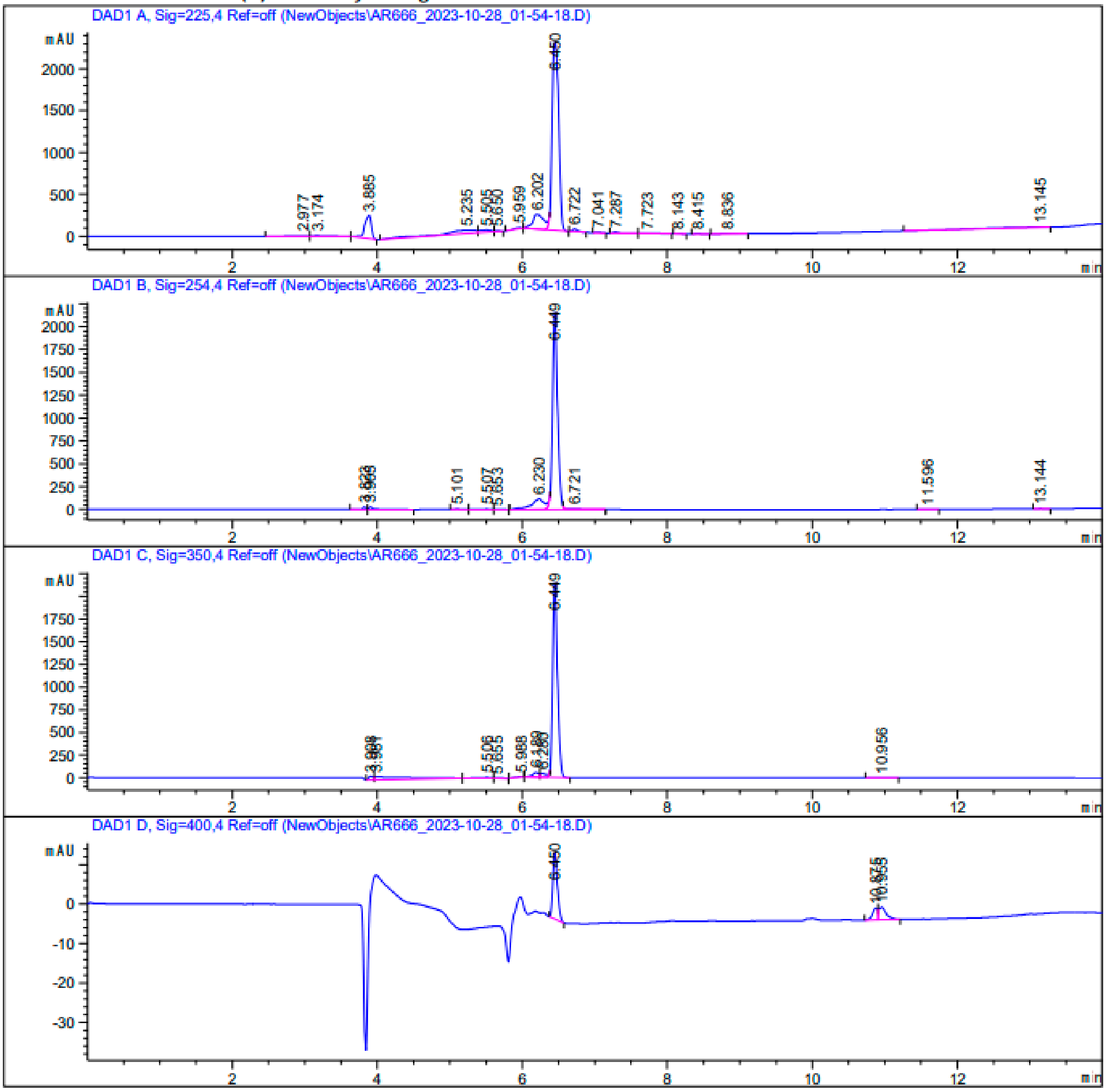

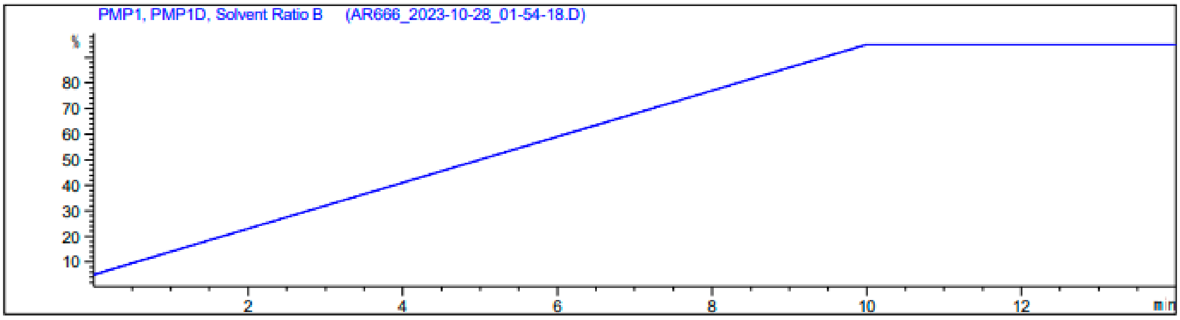

===
Fraction Information
===
No Fractions found.
===
===
===
Area Percent Report
===

Sorted By : Signal
Multiplier : 1.0000
Dilution : 1.0000
Use Multiplier & Dilution Factor with ISTDs

Signal 1: DAD1 A, Sig=225,4 Ref=off

Peak #	RetTime [min]	Type	Width [min]	Area [mAU*s]	Height [mAU]	Area %
1	2.977	BV	0.2628	99.11498	5.99675	0.4811
2	3.174	VB	0.1899	83.97901	6.28526	0.4076
3	3.885	BB	0.1006	1705.00793	273.82925	8.2753
4	5.235	BV	0.5132	1406.82581	37.88784	6.8280
5	5.505	VV	0.1412	301.27673	27.63102	1.4622
6	5.650	VB	0.0648	48.48973	10.72404	0.2353
7	5.959	BB	0.1134	85.03596	11.89835	0.4127
8	6.202	BV E	0.1576	1951.46985	175.65384	9.4715
9	6.450	VB R	0.1009	1.40723e4	2253.41675	68.3000
10	6.722	BB	0.0759	160.08669	32.25499	0.7770
11	7.041	BB	0.0694	36.92891	8.37458	0.1792
12	7.287	BV	0.0766	67.64575	13.46694	0.3283
13	7.723	VB	0.2121	63.40644	3.97394	0.3077
14	8.143	BB	0.0734	8.19242	1.72479	0.0398
15	8.415	BB	0.0697	16.97690	3.68704	0.0824
16	8.836	BB	0.1497	55.53268	5.50305	0.2695
17	13.145	BB	1.0656	441.38321	4.94367	2.1423

Totals : 2.06037e4 2877.25210

Signal 2: DAD1 B, Sig=254,4 Ref=off

Peak #	RetTime [min]	Type	Width [min]	Area [mAU*s]	Height [mAU]	Area %
1	3.823	BB	0.0431	81.98722	30.79661	0.6583
2	3.905	BB	0.0827	172.45445	29.28654	1.3847
3	5.101	BV	0.0885	39.91862	6.60172	0.3205
4	5.507	VV	0.1426	80.60844	7.31288	0.6472
5	5.653	VB	0.1087	53.89558	6.63917	0.4327
6	6.230	BV E	0.1694	1609.06543	122.36877	12.9198
7	6.449	VV R	0.0734	1.02503e4	2158.45264	82.3039
8	6.721	VB E	0.1849	129.64230	9.06448	1.0409
9	11.596	BB	0.0959	20.27358	3.29008	0.1628
10	13.144	BB	0.0951	16.06939	2.63518	0.1290
Totals :				1.24543e4	2376.44806	

Signal 3: DAD1 C, Sig=350,4 Ref=off

Peak #	RetTime [min]	Type	Width [min]	Area [mAU*s]	Height [mAU]	Area %
1	3.908	BV	0.0723	202.44543	41.91726	1.6889
2	3.981	VB	0.4313	1173.63867	33.28838	9.7912
3	5.506	BV	0.1394	95.92249	8.92333	0.8002
4	5.655	VB	0.1047	69.00599	8.87369	0.5757
5	5.988	BB	0.1843	47.39679	3.58452	0.3954
6	6.189	BV E	0.0869	302.65848	49.83351	2.5250
7	6.280	VV E	0.0852	254.17137	41.63456	2.1205
8	6.449	VB R	0.0712	9828.66504	2155.34668	81.9967
9	10.956	BB	0.1618	12.75899	1.08033	0.1064
Totals :				1.19867e4	2344.48226	

Signal 4: DAD1 D, Sig=400,4 Ref=off

Peak #	RetTime [min]	Type	Width [min]	Area [mAU*s]	Height [mAU]	Area %
1	6.450	BB	0.0665	72.96505	16.85627	65.5295
2	10.875	BV	0.0776	15.03281	2.94193	13.5009
3	10.955	VB	0.1072	23.34906	3.19538	20.9697
Totals :				111.34692	22.99357	

Ditrifluoroacetate salt of N-(2-guanidinoethyl)-2-(7-(2-((2-guanidinoethyl)amino)-2-oxoethoxy)-1-oxobenzo[4,5]imidazo[1,2-c]pyrimidin-2(1H)-yl)acetamide **7a**

```
=====================================================================
Acq. Operator   : SYSTEM
Sample Operator : SYSTEM
Acq. Instrument : Infinity II                   Location :   P1-B-01
Injection Date  : 28/10/2023 12:01:21 AM
                                              Inj Volume : 100.000 µl
Acq. Method     : C:\Chem32\1\Methods\New Objects.M
Last changed    : 27/10/2023 11:55:18 PM by SYSTEM
                  (modified after loading)
Analysis Method : C:\Chem32\1\Methods\Steps.M
Last changed    : 27/10/2023 8:41:47 PM by SYSTEM
                  (modified after loading)
Additional Info : Peak(s) manually integrated
```

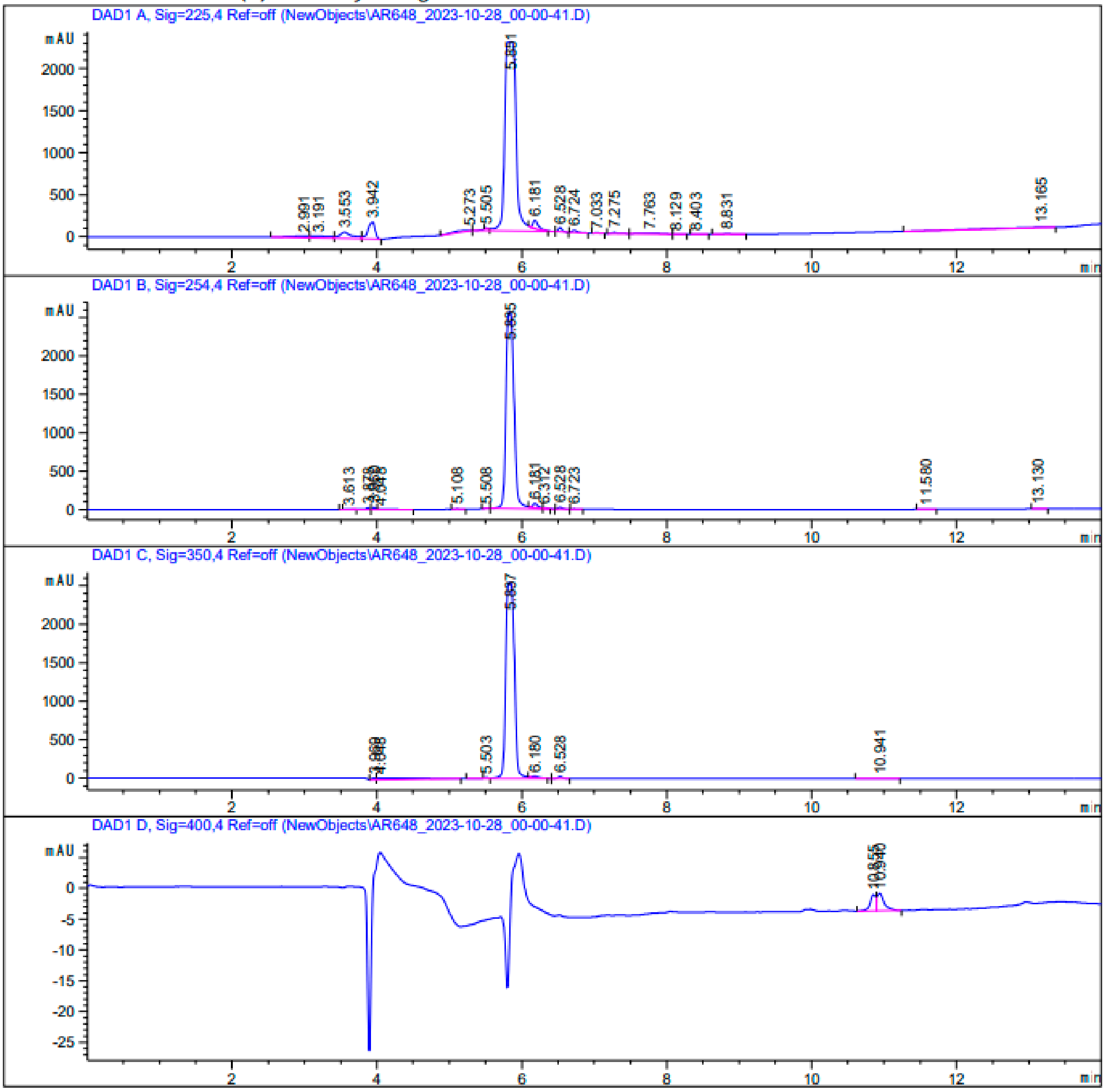

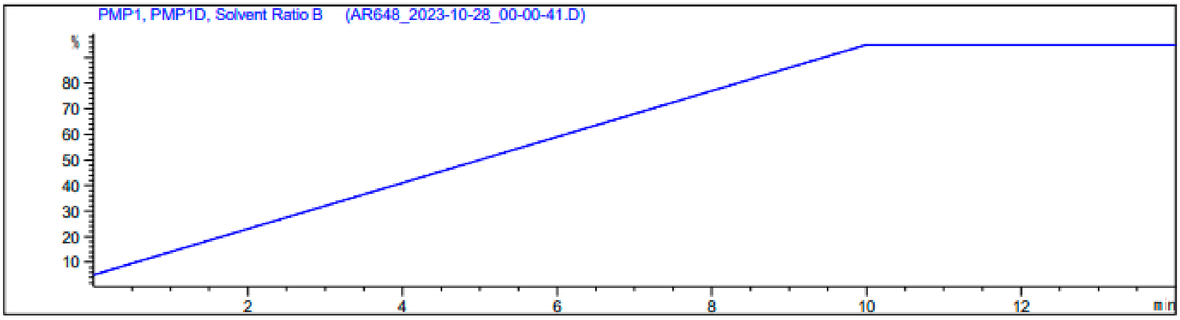

===
Fraction Information
===

No Fractions found.

===

===

===
Area Percent Report
===

Sorted By : Signal
Multiplier : 1.0000
Dilution : 1.0000
Use Multiplier & Dilution Factor with ISTDs

Signal 1: DAD1 A, Sig=225,4 Ref=off

Peak #	RetTime [min]	Type	Width [min]	Area [mAU*s]	Height [mAU]	Area %
1	2.991	BV	0.2799	287.88629	16.31576	0.9722
2	3.191	VV	0.2686	409.99109	20.92004	1.3845
3	3.553	VV	0.1699	909.50354	73.75916	3.0713
4	3.942	VB	0.1101	1398.02808	203.68585	4.7210
5	5.273	BB	0.5394	278.25024	6.15508	0.9396
6	5.505	BV E	0.0384	24.96999	11.01405	0.0843
7	5.851	VV R	0.1535	2.46378e4	2253.84058	83.1997
8	6.181	VB E	0.0711	480.10464	98.12334	1.6213
9	6.528	BB	0.0614	201.37849	51.70769	0.6800
10	6.724	BB	0.0745	134.77974	27.80754	0.4551
11	7.033	BB	0.0648	27.81246	6.64463	0.0939
12	7.275	BV	0.0906	77.87965	12.17313	0.2630
13	7.763	VV	0.3100	195.61382	9.35357	0.6606
14	8.129	VB	0.0846	12.89802	2.26074	0.0436
15	8.403	BB	0.0724	15.15675	3.13708	0.0512
16	8.831	BB	0.1455	52.53346	5.30547	0.1774
17	13.165	BB	0.8481	468.24521	6.89654	1.5812

Totals : 2.96128e4 2809.10026

Signal 2: DAD1 B, Sig=254,4 Ref=off

Peak #	RetTime [min]	Type	Width [min]	Area [mAU*s]	Height [mAU]	Area %
1	3.613	BV E	0.0869	7.23587	1.30036	0.0349
2	3.878	VB R	0.0575	96.65228	24.80041	0.4658
3	3.960	BV R	0.0528	91.24999	27.40560	0.4398
4	4.048	VB E	0.1830	53.74589	3.80063	0.2590
5	5.108	BB	0.0726	28.32966	6.05174	0.1365
6	5.508	BB	0.0519	26.92662	8.28064	0.1298
7	5.835	BV R	0.1250	2.00138e4	2565.58252	96.4574
8	6.181	VV E	0.0670	256.60352	56.51931	1.2367
9	6.312	VB E	0.0544	38.25103	10.51667	0.1844
10	6.528	BB	0.0616	90.08223	23.05393	0.4342
11	6.723	BB	0.0681	13.16357	3.06517	0.0634
12	11.580	BB	0.0895	18.08248	3.12718	0.0871
13	13.130	BB	0.0894	14.73012	2.55129	0.0710
Totals :				2.07489e4	2736.05544	

Signal 3: DAD1 C, Sig=350,4 Ref=off

Peak #	RetTime [min]	Type	Width [min]	Area [mAU*s]	Height [mAU]	Area %
1	3.969	BV	0.0637	101.59981	25.95711	0.4540
2	4.048	VB	0.4505	936.20721	25.60855	4.1839
3	5.503	BV E	0.0464	26.01128	9.37426	0.1162
4	5.837	VV R	0.1346	2.10973e4	2548.22485	94.2833
5	6.180	VB E	0.0727	105.91026	21.05140	0.4733
6	6.528	VB R	0.0616	96.38177	23.63672	0.4307
7	10.941	BB	0.1659	13.10032	1.01946	0.0585
Totals :				2.23765e4	2654.87236	

Signal 4: DAD1 D, Sig=400,4 Ref=off

Peak #	RetTime [min]	Type	Width [min]	Area [mAU*s]	Height [mAU]	Area %
1	10.855	BV	0.0779	14.11893	2.65911	41.0910
2	10.940	VB	0.1024	20.24125	2.86687	58.9090
Totals :				34.36019	5.52598	

Ditrifluoroacetate salt of N-(2-guanidinoethyl)-2-(5-(2-((2-guanidinoethyl)amino)-2-oxoethoxy)-11-oxonaphtho[2',1':4,5]imidazo[1,2-c]pyrimidin-10(11H)-yl)acetamide **7b**

```
=====================================================================
Acq. Operator   : SYSTEM
Sample Operator : SYSTEM
Acq. Instrument : Infinity II                     Location :   1
Injection Date  : 27/10/2023 10:51:53 PM
                                                Inj Volume : 50.000 µl
Acq. Method     : C:\Chem32\1\Methods\New Objects.M
Last changed    : 27/10/2023 10:50:02 PM by SYSTEM
                  (modified after loading)
Analysis Method : C:\Chem32\1\Methods\Steps.M
Last changed    : 27/10/2023 8:41:47 PM by SYSTEM
                  (modified after loading)
Additional Info : Peak(s) manually integrated
```

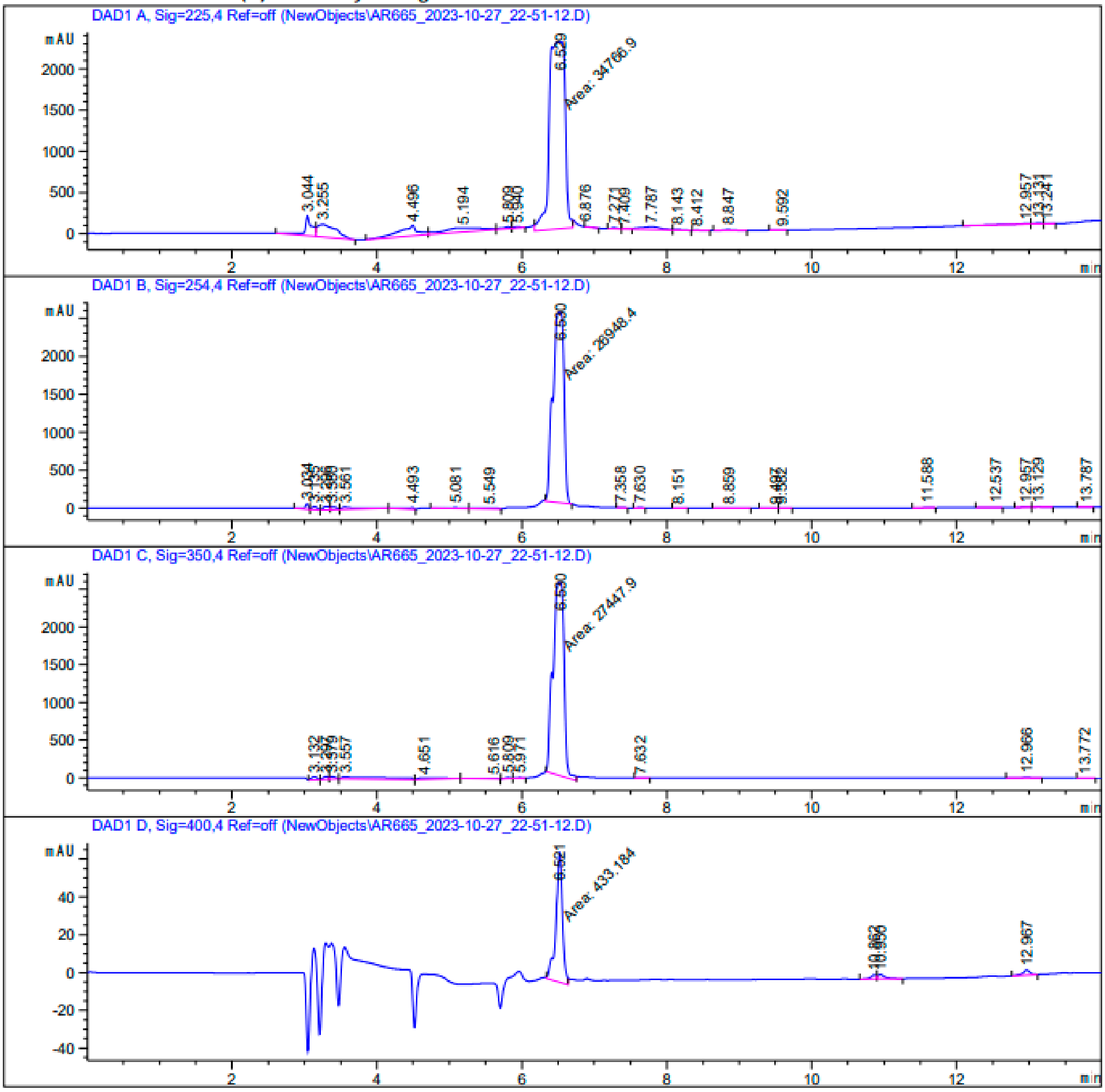

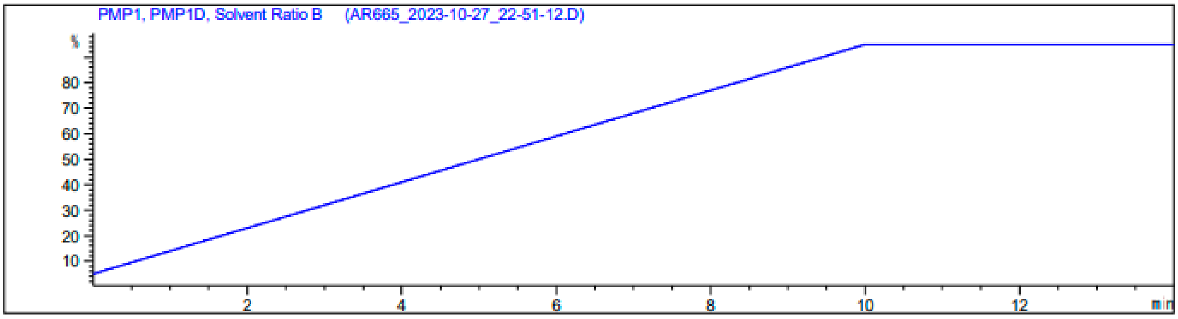

==

Fraction Information

==

No Fractions found.

==

==

===

Area Percent Report

===

Sorted By : Signal

Multiplier : 1.0000

Dilution : 1.0000

Use Multiplier & Dilution Factor with ISTDs

Signal 1: DAD1 A, Sig=225,4 Ref=off

Peak #	RetTime [min]	Type	Width [min]	Area [mAU*s]	Height [mAU]	Area %
1	3.044	BV	0.0952	1691.46558	237.23346	3.7575
2	3.255	VB	0.2489	2753.06641	150.83430	6.1158
3	4.496	BV	0.2195	2295.29541	129.02312	5.0989
4	5.194	VV	0.5562	1922.28210	49.15940	4.2702
5	5.809	VV	0.1204	202.85837	23.11438	0.4506
6	5.940	VB	0.1153	121.48501	15.18548	0.2699
7	6.529	MM	0.2560	3.47669e4	2263.74585	77.2325
8	6.876	BB	0.1483	17.22005	1.49560	0.0383
9	7.271	BV	0.0872	74.71729	12.59482	0.1660
10	7.409	VV E	0.0678	8.35291	1.81164	0.0186
11	7.787	VV R	0.2825	683.26587	33.70721	1.5178
12	8.143	VB	0.0971	50.52599	7.84774	0.1122
13	8.412	BB	0.0806	14.25985	2.57339	0.0317
14	8.847	BB	0.1189	78.36713	9.24578	0.1741
15	9.592	BB	0.0985	9.66174	1.40081	0.0215
16	12.957	BV	0.2911	206.05789	8.86771	0.4577
17	13.131	VV	0.1089	85.73798	11.25253	0.1905
18	13.241	VB	0.0838	34.34385	6.08851	0.0763

Totals : 4.50158e4 2965.18175

Signal 2: DAD1 B, Sig=254,4 Ref=off

Peak #	RetTime [min]	Type	Width [min]	Area [mAU*s]	Height [mAU]	Area %
1	3.034	BV	0.0502	230.93816	70.50156	0.8001
2	3.135	VB	0.0949	202.21748	36.35234	0.7006
3	3.296	BV	0.0917	257.77933	43.12821	0.8930
4	3.380	VV	0.0895	192.25668	35.33190	0.6660
5	3.561	VB	0.2107	429.07809	27.09868	1.4865
6	4.493	BB	0.1079	182.57790	21.76458	0.6325
7	5.081	BV	0.1594	110.89911	9.14938	0.3842
8	5.549	VB	0.2634	175.43741	8.20821	0.6078
9	6.530	MM	0.1793	2.69484e4	2505.38501	93.3594
10	7.358	BB	0.0749	8.40835	1.78682	0.0291
11	7.630	BB	0.0655	5.34977	1.26165	0.0185
12	8.151	BB	0.0820	5.66673	1.10096	0.0196
13	8.859	BB	0.1580	12.76490	1.09534	0.0442
14	9.497	BV	0.0735	6.17648	1.21171	0.0214
15	9.582	VB	0.0772	5.33936	1.01787	0.0185
16	11.588	BB	0.0918	11.63050	1.94496	0.0403
17	12.537	BB	0.1226	12.03319	1.36821	0.0417
18	12.957	BV	0.0898	17.93754	3.00014	0.0621
19	13.129	VB	0.0969	41.75738	6.33346	0.1447
20	13.787	VB	0.1104	8.59167	1.13338	0.0298
Totals :				2.88653e4	2778.17437	

Signal 3: DAD1 C, Sig=350,4 Ref=off

Peak #	RetTime [min]	Type	Width [min]	Area [mAU*s]	Height [mAU]	Area %
1	3.132	BB	0.0995	242.70677	43.23568	0.7991
2	3.297	BV	0.0854	215.77141	39.72534	0.7104
3	3.379	VB	0.0924	199.10532	34.97945	0.6556
4	3.557	BB	0.6003	1455.89368	29.57506	4.7936
5	4.651	BB	0.3014	434.84229	20.02106	1.4317
6	5.616	BB	0.2266	171.16843	9.85601	0.5636
7	5.809	BV	0.1076	94.60773	14.22901	0.3115
8	5.971	VB	0.1322	53.38142	5.86212	0.1758
9	6.530	MM	0.1788	2.74479e4	2558.37500	90.3727
10	7.632	BB	0.0708	6.84686	1.40538	0.0225
11	12.966	BB	0.1021	37.89906	5.38847	0.1248
12	13.772	BBA	0.0923	11.76891	1.95303	0.0387
Totals :				3.03719e4	2764.60562	

Signal 4: DAD1 D, Sig=400,4 Ref=off

Peak #	RetTime [min]	Type	Width [min]	Area [mAU*s]	Height [mAU]	Area %
1	6.521	MM	0.1051	433.18408	68.70710	90.8770
2	10.862	BV	0.0761	10.85815	2.10665	2.2779
3	10.950	VB	0.0976	15.70599	2.30270	3.2949
4	12.967	BB	0.0968	16.92251	2.56995	3.5501
Totals :				476.67073	75.68639	
